# Adjacent Neuronal Fascicle Guides Motoneuron 24 Dendritic Branching and Axonal Routing Decisions through Dscam1 Signaling

**DOI:** 10.1523/ENEURO.0130-24.2024

**Published:** 2024-10-18

**Authors:** Kathy Clara Bui, Daichi Kamiyama

**Affiliations:** Department of Cellular Biology, University of Georgia, Athens, Georgia 30605

**Keywords:** Axon, CNS, dendrite, *Drosophila*, Dscam, motoneuorn

## Abstract

The formation and precise positioning of axons and dendrites are crucial for the development of neural circuits. Although juxtacrine signaling via cell–cell contact is known to influence these processes, the specific structures and mechanisms regulating neuronal process positioning within the central nervous system (CNS) remain to be fully identified. Our study investigates motoneuron 24 (MN24) in the *Drosophila* embryonic CNS, which is characterized by a complex yet stereotyped axon projection pattern, known as “axonal routing.” In this motoneuron, the primary dendritic branches project laterally toward the midline, specifically emerging at the sites where axons turn. We observed that Scp2-positive neurons contribute to the lateral fascicle structure in the ventral nerve cord (VNC) near MN24 dendrites. Notably, the knockout of the Down syndrome cell adhesion molecule (*Dscam1*) results in the loss of dendrites and disruption of proper axonal routing in MN24, while not affecting the formation of the fascicle structure. Through cell-type specific knockdown and rescue experiments of Dscam1, we have determined that the interaction between MN24 and Scp2-positive fascicle, mediated by Dscam1, promotes the development of both dendrites and axonal routing. Our findings demonstrate that the holistic configuration of neuronal structures, such as axons and dendrites, within single motoneurons can be governed by local contact with the adjacent neuron fascicle, a novel reference structure for neural circuitry wiring.

## Significance Statement

We uncover a key neuronal structure serving as a guiding reference for neural circuitry within the *Drosophila* embryonic CNS, highlighting the essential role of an adjacent axonal fascicle in precisely coordinating axon and dendrite positioning in motoneuron 24 (MN24). Our investigation of cell–cell interactions between motoneurons and adjacent axonal fascicles—crucial for initiating dendrite formation, soma mislocation, and axonal pathfinding in MN24—emphasizes the neuronal fascicle’s significance in neural circuit formation through Dscam1-mediated interneuronal communication. This enhances our understanding of the molecular underpinnings of motoneuron morphogenesis in *Drosophila*. Given the occurrence of analogous axon fascicle formations within the vertebrate spinal cord, such structures may play a conserved role in the morphogenesis of motoneurons via Dscam1 across phyla.

## Introduction

The positioning of axons and dendrites is crucial for neuronal function (Yogev and Shen, 2014, 2017; Lefebvre et al., 2015; Lanoue and Cooper, 2019), particularly during early embryonic stages when neural circuits are formed independently of neuronal activities, under the guidance of the developmental program (Haverkamp, 1986; Verhage et al., 2000; Varoqueaux et al., 2002; Constance et al., 2018). Extracellular cues play a critical role in neural circuit development by providing spatial information that guides the growth and patterning of neural structures (Sanes, 1989; Guan and Rao, 2003; Long and Huttner, 2019). A well-studied example of spatial regulation in neuronal processes is seen in the decision-making regarding repulsion and attraction in reference to the midline within the embryonic central nervous system (CNS) of *Drosophila melanogaster* (Klambt et al., 1991; Evans and Bashaw, 2010; Howard et al., 2019). In the CNS, neurons can project their axons across the midline to the contralateral side or stay on the ipsilateral side (Kaprielian et al., 2001). This critical decision relies on the diffusible midline ligands like Slit and Netrin binding with their respective receptors Roundabout (Robo) and Frazzled (Fra) expressed on axons (Kidd et al., 1998, 1999; Brose et al., 1999; Harris et al., 1996; Hiramoto et al., 2000). Similarly, these signaling mechanisms also direct the later, higher-order branches of dendrites in the CNS (Furrer et al., 2003, 2007; Mauss et al., 2009). However, it remains to be determined whether these diffusible molecules from the midline exclusively regulate how neuronal processes are directed to their proper destinations within the embryonic CNS.

Current research identifies only a few adhesion molecules crucial for juxtacrine signaling in this context (Howard et al., 2019). For example, the atypical cadherin Flamingo is involved in axon midline crossing (Organisti et al., 2015), while Down syndrome cell adhesion molecule (Dscam1) promotes axon growth across segments (Alavi et al., 2016). Yet, their roles in CNS dendrite formation are largely unexplored. Our previous study fills this gap by demonstrating Dscam1’s significant role in dendritic outgrowth (Kamiyama et al., 2015). We investigated anterior corner cell (aCC) motoneurons, focusing on their lateral axonal extensions and interactions with MP1 partner neurons. We discovered that dendritogenesis is initiated by a cell–cell adhesion mechanism facilitated by Dscam1 interactions, where Dscam1 on one neuron binds to another, leading to cytoskeletal changes and dendritic growth in aCC motoneuron. The extent to which this Dscam1-mediated mechanism is generalizable among motoneurons, and its broader impact on *Drosophila* neuronal morphology, warrants further exploration. Additionally, evidence suggests Dscam1’s involvement in processes like cell body migration, axon guidance, and dendrite patterning, underscoring its broad involvement in neural development (Schmucker et al., 2000; Wojtowicz et al., 2004; Zhan et al., 2004; Zhu et al., 2006; Goyal et al., 2019; Liu et al., 2020; Dong et al., 2022; Wilhelm et al., 2022).

In the embryonic CNS, 36 motoneurons per hemisegment have been mapped (Sink and Whitington, 1991; Landgraf et al., 1997). These motoneurons are categorized into two groups: the first with cell bodies situated between the midline and the neuropiles along the mediolateral plane and the second with cell bodies located outside the neuropiles, extending to the edge of the ventral nerve cord (VNC). The aCC motoneuron falls into this former category. In our current investigation, we shift our focus to the latter category to further explore Dscam1-mediated neural morphogenesis. Motoneurons in this second category exhibit an axon turning pattern and elaborate their dendrites specifically at these axonal turning points. One such motoneuron, MN24, displays a unique pattern of dendritic elaboration. Contrary to the aCC motoneuron, whose dendritic arbors are in the middle region of the neuropile, MN24’s dendritic projections are predominantly observed at the most lateral edge of the neuropile, without overlapping aCC processes. This positioning makes MN24 an ideal model for investigating the molecular and cellular mechanisms underlying axon turning and dendritic arborization in identifiable single motoneurons within the CNS.

We have anatomically characterized MN24 and its potential partner, a Scp2-positive neuronal fascicle. Our studies demonstrate that a *Dscam1* knockout eliminates dendrites and disrupts MN24 axonal routing. Cell-specific *Dscam1* manipulations reveal that Dscam1-mediated contact between MN24 and Scp2-positive neurons is essential for both dendrite and axon development. These findings introduce a novel fascicle structure in which neuronal morphology is modulated through Dscam1 adhesion.

## Materials and Methods

### Fly stocks

Canton-S was used as a wild-type strain (source: W. Kim). For mutant analyses, *Dscam1^21^* (source: J. Wang) was used. The following lines were obtained from the Bloomington *Drosophila* Stock Center: *UAS*-*mCD4::tdGFP* (#35836), *UAS*-*mCD4::tdTomato* (#35841), *UAS*-*Dscam1 RNAi* (#38945), *hh*-GAL4 (#49437), *scp2*-GAL4 (#49538), *UAS*-*MCFO-1* (#64085), *UAS-hs-FLPG5* (#58356), *UAS- 10xUAS(FRT.stop)myr::smGdP-V5-THS-10xUAS(FRT.stop)myr::smGdP-FLAG* (#62123), and *Kr*-*GFP* balancer (#5195). Homozygous mutants were identified using GFP balancers. *hh-GAL4* and *Scp2-GAL4* were used for transgenic expression in MN24 and a subset of the lateral fascicle, respectively, from the Janelia GAL4 stocks. For the rescue experiments in *Dscam1^−/−^*, in Figures 4*C*, 6*C*, and 7*A*, a single isoform of *Dscam1* (*UAS-Dscam1^exon 17.2^-GFP*; source: T. Lee) was used. Specific fly genotypes in experiments are described in Table 1. Flies were reared at 25°C using standard procedures.

**Table 1.** Genotypes of flies shown in this study, related to the figures and extended data figures

Figure	Genotype
Figure 1*A*,*B*	*w;+/+*
Figure 2*A*	*w;+/+*
	*w*;*Dscam1^21^/Dscam1^21^*(*Dscam1−/−*)
Figure 2*D*	*w;+/+*
	*w*;*Dscam1^21^/Dscam1^21^*(*Dscam1−/−*)
Figure 2*F*	*w;+/+*
	*w*;*Dscam1^21^/Dscam1^21^*(*Dscam1−/−*)
Figure 3*A–C*	*hsFLP*/+; *UAS-MCFO-1*/*scp2-GAL4* (control)
Figure 3*D–F*	*w*;*Dscam1^21^/Dscam1^21^*; *hsFLPG5*/*scp2-GAL4, UAS-MCFO-1* (*Dscam1^−/−^*)
Figure 4*A*	*w;;hh-GAL4/UAS-mCD4::tdGFP* (positive control)
	*w;;hh-GAL4/UAS-Dscam1 RNAi* (*hh-GAL4 UAS-Dscam1 RNAi*)
Figure 4*C*	*w*;*Dscam1^21^/Dscam1^21^*;*hh-GAL4/UAS-mCD4::tdGFP* (negative control)
	*w*;*Dscam1^21^/Dscam1^21^*; *UAS-mCD4::tdGFP*/*hh-GAL4, UAS-Dscam1^17.2^::GFP* (*hh-GAL4 UAS-Dscam1*)
Figure 5*A–C*	*w;;scp2-GAL4/UAS-mCD4::tdGFP*
Figure 6*A*	*w;;scp2-GAL4/UAS-mCD4::tdGFP* (positive control)
	*w;;scp2-GAL4/UAS-Dscam1 RNAi* (*scp2-GAL4 UAS*-*Dscam1 RNAi*)
Figure 6*C*	*w*;*Dscam1^21^/Dscam1^21^*;*scp2-GAL4/UAS-mCD4::tdGFP* (negative control)
	*w*;*Dscam1^21^/Dscam1^21^*; *UAS-Dscam1^17.2^::GFP*/*scp2-GAL4, UAS-CD4::tdGFP* (*scp2-GAL4 UAS-Dscam1*)
Figure 7	*w;;scp2-GAL4, UAS-CD4::tdGFP/hh-GAL4* (positive control)(top)
	*w*;*Dscam1^21^/Dscam1^21^*; *scp2-GAL4, UAS-mCD4::tdGFP/hh-GAL4*(negative control; middle)
	*w*;*Dscam1^21^/Dscam1^21^*; *scp2-GAL4, UAS-mCD4::tdGFP/hh-GAL4, UAS-Dscam1^17.2^::GFP* (*hh-GAL4*& *scp2-GAL4 UAS-Dscam1*; bottom)
Extended Data Figure 1	*w;+/+*
Extended Data Figure 6*A*	*w;;scp2-GAL4/UAS-Dscam1 RNAi* (*scp2-GAL4 UAS*-*Dscam1 RNAi*)
Extended Data Figure 6*B*	*w*;*Dscam1^21^/Dscam1^21^*;*scp2-GAL4/UAS-mCD4::tdGFP* (*Dscam1^−/−^*)
Extended Data Figure 6*C*	*w;;scp2-GAL4/UAS-mCD4::tdGFP* (control; top)
	*w*;*Dscam1^21^/Dscam1^21^*;*scp2-GAL4/UAS-mCD4::tdGFP* (*Dscam1^−/−^*; bottom)

The genotypes used in this study are detailed in this table.

### RNAi experiments

For cell-specific RNAi experiments, the *UAS-shRNA* line that targets all splice variants known for *Dscam1* was obtained from TRiP at Harvard Medical School via BDSC. For examinations of *Dscam1* functions, in Figures 4*A* and 6*A*, the *Dscam1* RNAi construct was expressed in various small subsets of neurons (*UAS-mCD4::tdTomato*/+;*UAS-Dscam1RNAi*/*GAL4*). Detailed information on used *GAL4* drivers is listed in Table 1.

### Immunohistochemistry

Embryos were fillet dissected, fixed with 4% paraformaldehyde for 5 min, and blocked in a solution of PBS/0.01% Triton X-100 with 0.06% BSA (TBSB) for 1 h at room temperature (RT). For labeling of Scp2-positive lateral fascicle with a reference pattern [anti-Fasciclin II (FasII) and/or anti-Horseradish Peroxidase (HRP)], the embryos were incubated with anti-FasII (mouse mAb, Developmental Studies Hybridoma Bank at 1:500) in TBSB at 4°C overnight. For single MN24 labeling using Multi-Color Flip Out (MCFO), the embryos were incubated with anti-FasII at 1:100 and anti-FLAG (rat mAb, Novus Biologicals at 1:50) in TBSB at 4°C overnight. Samples were washed three times for 5 min with TBSB and incubated with conjugated anti-HRP (goat mAb, Jackson ImmunoResearch; 1:500) and secondary antibodies (Alexa Fluor 647 Donkey anti-mouse, Invitrogen at 1:500; and/or Alexa Fluor 555 Donkey anti-rat, Thermo Fisher Scientific at 1:500) for 2 h at RT and washed with PBS. Anti-HRP conjugation with fluorescent dyes was performed by following the same procedure as described in previous literature (Inal et al., 2021). Following immunohistochemistry, samples were postfixed with 4% paraformaldehyde for 10 min and mounted in PBS.

### Fluorescence imaging

Confocal microscopy images of fillet embryos expressing green or red fluorescent proteins alongside far-red DiD-labeled neurons were captured using an inverted fluorescence microscope (Ti-E, Nikon) with either 40× 0.80 NA water immersion objective or 100× 1.45 NA oil immersion objective (Nikon). The microscope was attached to the Dragonfly Spinning disk confocal unit (CR-DFLY-501, Andor). Three excitation lasers (40 mW 488 nm, 50 mW 561 nm, and 110 mW 642 nm lasers) were coupled to a multimode fiber passing through the Andor Borealis unit. A dichroic mirror (Dragonfly laser dichroic for 405-488-561-640) and three bandpass filters (525/50 nm, 600/50 nm, and 725/40 nm bandpass emission wheel filters) were placed in the imaging path. Images were recorded with an electron-multiplying charge-coupled device camera (iXon, Andor).

### Labeling dendrites and quantifying dendritic processes in MN24

For phenotypic analyses of dendritic processes in wild-type and mutant backgrounds, DiD labeling (Thermo Fisher Scientific) of MN24 was performed in embryos at 15:00 or 18:00 AEL by following the same procedure as described in the literature (Inal et al., 2020). To minimize the variation in the dendritic processes in MN24 in different segments, neurons from abdominal segments 2–7 were imaged. Primary dendritic processes in individual MN24 that were longer than 1.0 µm were counted.

### Quantitative measurement of MN24 cell body position

For phenotypic analyses of the cell body position in a wild-type background, single MN24 neurons were labeled in 9:00 AEL and 12:00 AEL embryos by crossing a Multi-Color Flip Out (MCFO) fly line containing a heat shock-inducible FLP recombinase construct with the *hh-GAL4* line. For mutant analyses, the same genetic cross was performed under the *Dscam1^21^* null background. A heat shock (37°C) was applied for 30 min to embryos aged 6:00–10:00 AEL on grape juice agar. Embryos were then incubated at room temperature (23°C) for at least 2 h before dissection. Typically, a couple of MN24 neurons per embryo were fluorescently labeled. After imaging, the distances from the center of mass of individual cell bodies to the midline were measured.

MCFO was chosen over other flip-out techniques because of a membrane labeling approach optimized for fine neuronal processes, called “spaghetti monster GFP (smGFP)” (Nern et al., 2015). smGFP consists of HA, V5, and FLAG epitopes, with FLAG showing the best performance in embryos.

### Quantitative measurement of MN24 and Scp2-Positive lateral fascicle position

MN24 was labeled with DiD and genetically encoded membrane markers (mCD4::tdGFP or mCD4::tdTomato). Similarly, Scp2-positive lateral fascicles are marked using the aforementioned genetically encoded membrane markers. Confocal stacks were acquired varying between 0.1 and 0.5 µm *z*-steps. The distance of the FasII- or Scp2-positive lateral fascicle from the midline was measured by first generating the FasII- or Scp2-positive lateral fascicle intensity profile perpendicular to the midline. Then, measurements are fitted in a histogram plot. Images were analyzed using Fiji. The figures were prepared using Adobe Illustrator and Photoshop.

### Experimental design and statistical analyses

A between-subject design was employed in all experiments. Immunohistochemistry and dye-labeling experiments were repeated at least two and 10 times, respectively, using flies from independent crosses. Statistical analyses were performed and visualized using JMP Pro 16. The results of the statistical tests are shown in Table 2. All datasets were assessed for normality using Shapiro–Wilk’s test, and nonparametric tests were employed when the normality assumption was not met. Comparisons between two groups were analyzed using nonparametric Mann–Whitney *U* test or parametric Welch’s *t* test and Student’s *t* test. Comparisons between multiple groups were analyzed using nonparametric Kruskal–Wallis test. Post hoc comparisons were performed using Dunn’s multiple-comparison test. Error bars are shown as the standard error of the mean (SEM) in the figures.

**Table 2.** Statistical analyses grouped by figure number and panel and statistical tests

Figure	Test					
	Mann–Whitney	*n*1	*n*2	Exact *p* value	*U*	
2*B*, dendrite count	WT versus *Dscam1^−/−^*	22	15	<0.0001	120	
2*C*, axon routing	WT versus *Dscam1^−/−^*	22	15	<0.0001	131	
4*B*, dendrite count	*hh* > + versus *hh *> *Dscam1 RNAi*	12	14	<0.0001	268	
4*E*, axon routing	*hh* > + versus *hh *> *Dscam1 RNAi*	12	14	0.0017	242	
	Kruskal–Wallis test followed by Dunn’s multiple-comparisons tests	*n*1	*n*2	Adjusted *p* value	*Z*	
4*D*, dendrite count	*hh* > + in WT background versus *hh > + *in *Dscam1^−/−^* background	12	9	0.0002	4.01908	
	*hh* > + in *Dscam1^−/−^* background versus *hh > Dscam1* in *Dscam1^−/−^* background	9	16	1.0000	0.87597	
	*hh* > + in WT background versus *hh > Dscam1* in *Dscam1^−/−^* background	12	16	0.0007	3.66973	
4*F*, axon routing	*hh* > + in WT background versus *hh > + *in *Dscam1^−/−^* background	12	9	0.0012	3.53773	
	*hh* > + in *Dscam1^−/−^* background versus *hh > Dscam1* in *Dscam1^−/−^* background	9	16	1.0000	0.25691	
	*hh* > + in WT background versus *hh > Dscam1* in *Dscam1^−/−^* background	12	16	0.0005	3.78933	
	Mann–Whitney	*n*1	*n*2	Exact *p* value	*U*	
6*B*, dendrite count	*scp2* > + versus *scp2 *> *Dscam1 RNAi*	12	19	<0.0001	304	
6*E*, axon routing	*scp2* > + versus *scp2 *> *Dscam1 RNAi*	12	19	0.0042	263	
	Kruskal–Wallis test followed by Dunn’s multiple-comparisons tests	*n*1	*n*2	Adjusted *p* value	*z*	
6*D*, dendrite count	*scp2* > + in WT background versus *scp2 > + *in *Dscam1^−/−^* background	12	17	0.0001	4.10278	
	*scp2* > + in *Dscam1^−/−^* background versus *scp2 > Dscam1* in *Dscam1^−/−^* background	17	16	0.7877	1.12036	
	*scp2* > + in WT background versus *scp2 > Dscam1* in *Dscam1^−/−^* background	12	16	<0.0001	5.08455	
6*F*, axon routing	*scp2* > + in WT background versus *scp2 > + *in *Dscam1^−/−^* background	12	17	<0.0001	4.54159	
	*scp2* > + in *Dscam1^−/−^* background versus *scp2 > Dscam1* in *Dscam1^−/−^* background	17	16	0.4691	1.41738	
	*scp2* > + in WT background versus *scp2 > Dscam1* in *Dscam1^−/−^* background	12	16	0.0044	3.1786	
	Kruskal–Wallis test followed by Dunn’s multiple-comparisons tests	*n*1	*n*2	Adjusted *p* value	*z*	
7*B*, dendrite count	*scp2,hh* > + in WT background versus *scp2,hh > + *in *Dscam1^−/−^* background	12	11	<0.0001	4.59036	
	*scp2,hh* > + in D*scam1^−/−^* background versus *scp2,hh > Dscam1* in *Dscam1^−/−^* background	11	14	0.0004	3.81124	
	*scp2,hh* > + in WT background versus *scp2,hh > Dscam1* in *Dscam1^−/−^* background	12	14	1.0000	0.95042	
7*C*, axon routing	*scp2,hh* > + in WT background versus *scp2,hh > + *in D*scam1^−/−^* background	12	11	0.0005	3.74981	
	*scp2,hh* > + in *Dscam1^−/−^* background versus *scp2,hh > Dscam1* in *Dscam1^−/−^* background	11	14	0.0003	3.90274	
	*scp2,hh* > + in WT background versus *scp2,hh > Dscam1* in *Dscam1^−/−^* background	12	14	1.0000	0.0000	
	Welch’s *t* test	*n*1	*n*2	Adjusted *p* value	*t*	DF
Extended Data 6D, fascicle to midline	*scp2* > + in WT background versus *scp2 > + *in *Dscam1^−/−^* background	12	17	0.1585	1.4505	26.937
Extended Data 6-1E, soma to midline	*scp2* > + in WT background versus *scp2 > + *in *Dscam1^−/−^* background	12	17	<0.0001	4.9343	25.317
	Kruskal–Wallis test followed by Dunn’s multiple-comparisons tests	*n*1	*n*2	Adjusted *p* value	*z*	
Extended Data 7-1, soma to midline	*scp2,hh* > + in WT background versus *scp2,hh > + *in *Dscam1^−/−^* background	12	11	<0.0001	4.24784	
	*scp2,hh* > + in D*scam1^−/−^* background versus *scp2,hh > Dscam1* in D*scam1^−/−^* background	11	14	0.0007	3.6843	
	*scp2,hh* > + in WT background versus *scp2,hh > Dscam1* in *Dscam1^−/−^* background	12	14	1.0000	0.71709	

Detailed statistical analyses, including sample sizes and related statistical values, are provided in this table.

## Results

### Spatial regulation of MN24 dendritogenesis in late embryonic CNS

**Figure 1.**
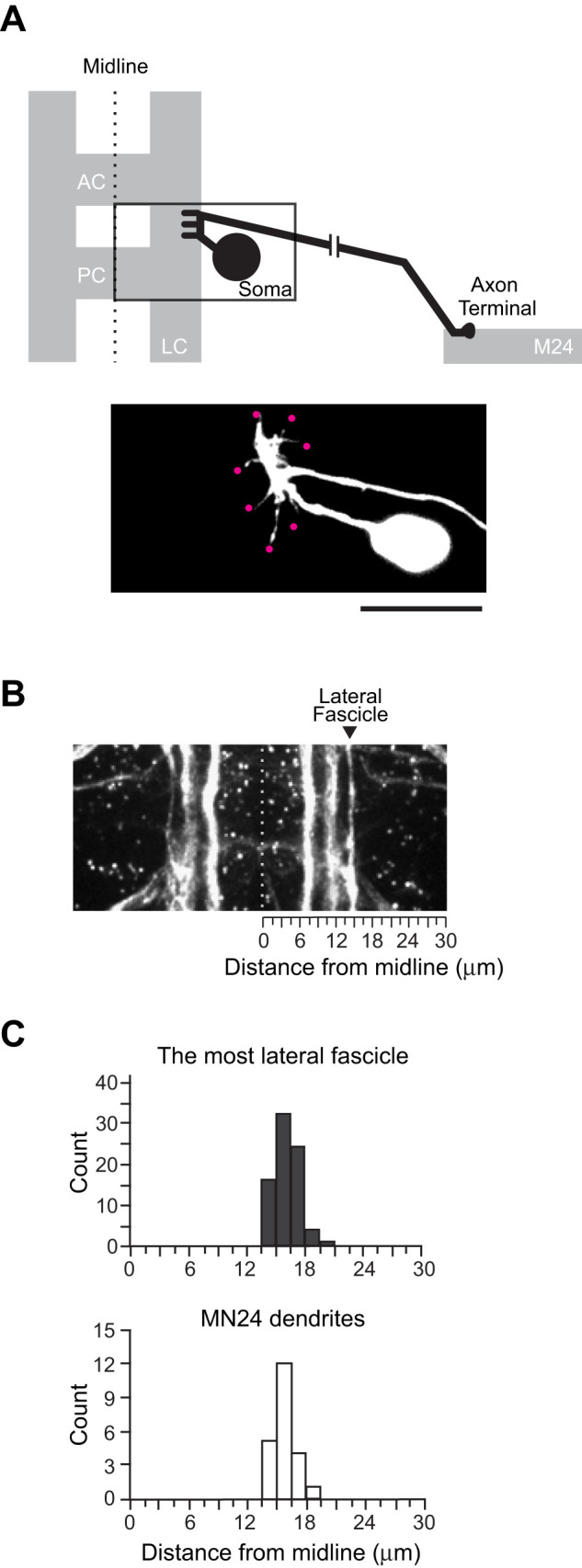
Neuronal fascicle spatially aligns with the position of MN24 dendrite formation. ***A***, Top panel shows a schematic of MN24 (black) within the ventral nerve cord of an embryo at 15:00 AEL—all subsequent images are taken at 15:00 AEL unless otherwise specified. The axon stereotypically projects out of the soma, anteriorly along the edge of the longitudinal connective (LC), and away from the midline to target muscle 24 (M24). The bottom panel shows a representative fluorescence image of a lipophilic dye-labeled MN24. At 15:00 AEL, MN24 form their dendritic processes (magenta dots) at stereotyped positions on the axon routing. For all subsequent images, anterior is to the top, and medial is to the left. AC, anterior commissure. PC, posterior commissure. Scale bar, 10 µm. ***B***, Representative fluorescence images of FasII-positive longitudinal fascicles within the ventral nerve cord. The stereotyped most lateral FasII-positive fascicle structure (arrowhead) provides a frame of reference to characterize the mediolateral position of MN24 dendrites. Gray dashed line depicts the midline. ***C***, Distribution plots of the mediolateral positions of MN24 dendritic branches (white; *n* = 22 neurons) and FasII-positive lateral fascicle (dark gray; *n* = 77 hemisegments), where 0 µm indicates the distance from the CNS midline. We indicate the location of the measured area for each MN24 axonal loop in Extended Data Figure 1-1.

Previous studies have demonstrated that the dendritic processes of the MN24 are distinctly positioned away from those in the aCC motoneuron, exhibiting a notable lateral shift (Landgraf et al., 1997, 2003b). While these studies have characterized the dendritic morphologies of MN24 qualitatively, there are no current reports that carefully examine the exact positioning and detailed arrangements of its dendritic branches. In our prior study (Kamiyama et al., 2015), we identified a MN24-specific GAL4 driver, *hedgehog (hh)-GAL4*, during a screening of GAL4 drivers. Notably, this *hh-GAL4* driver initiates GAL4 expression at 9:00 AEL in MN24, as well as in several neighboring motoneurons (MN21, MN22, and MN23). Initially, using this GAL4 driver, we attempted to label neuronal processes by expressing a membrane marker (*UAS-mCD4::tdGFP*). However, due to the low expression level of this GAL4 driver, we were unable to achieve adequate membrane labeling to distinguish fine neuronal structures. Consequently, we decided to employ a retrograde lipophilic dye-labeling technique. This method allowed us to label membranes with a high density of lipophilic dye, enabling the detailed visualization of individual dendritic branches (Fig. 1*A*). We then quantified the number and position of dendritic tips, the latter defined as the distance from the midline to the ventral nerve cord edge (Fig. 1*A*,*B*). These measurements reveal that on average, wild-type MN24 at 15:00 AEL has 7.6 ± 0.3 (mean ± SEM) primary dendritic branches, which are located 15.8 ± 0.3 µm from the midline (Fig. 1*A*,*C*). In addition to dendritic characteristics, we measured other anatomical features of MN24. The cell body of MN24 is located outside of the neuropile at 25.6 ± 0.8 µm from the midline. The axonal process of MN24 extends 7.9 ± 0.8 µm toward the midline before diverging away from it, forming a “routing” pattern. Following this divergence, the axon exits the CNS and then innervates the target muscle 24. Additionally, we quantified the area encompassed by the axonal routing, finding it to be 36.2 ± 3.6 µm^2^ (Extended Data Fig.1-1 for details of area measurement). Together, the arrangement of MN24 neuronal processes—specifically, its dendrite formation—is stereotypically positioned, suggesting MN24 dendrites are regulated in a spatial manner.

10.1523/ENEURO.0130-24.2024.f1-1 Figure 1-1 **Segment-specific MN24 morphologies in the Wild-Type Background** Representative images depicting the morphology of wild-type MN24 in different abdominal segments are shown. These images show the characteristic dendrites and axon routing, observed in Figure 1A. Notably, the angle of the axon segment projecting towards the muscle varies in a segment-specific manner. Axon routing area (shaded blue) is measured as the area within the loop. For “open” axon routing areas, (left and middle panels), we define the center of the cell body (purple dot) and use the perpendicular line to the soma center as the border for measurement of the axon routing area. Scale bar, 10 μm. Download Figure 1-1, TIF file.

### Potential guiding role of the most lateral fascicle structure to MN24 dendritogenesis

The emergence of primary dendritic branches of MN24 at specific lateral positions within the CNS prompts the following question: what spatial cue guides MN24 to generate its branches at the precise location? To understand the positioning of MN24 dendrites relative to established positional landmarks, we performed immunostaining on wild-type embryos using an anti-FasII antibody. This antibody reveals a set of axon tracts, each forming distinct longitudinal fascicles within the neuropile (Landgraf et al., 2003a). These tracts run along the anterior–posterior axis and parallel to each other in the mediolateral direction (Fig. 1*B*). Notably, the most lateral fascicle is located 16.2 ± 0.1 µm from the midline, closely mirroring the positioning of MN24 dendrites at 15.8 ± 0.3 µm (Fig. 1*C*). Due to the incompatibility between immunohistochemistry and lipophilic dye-labeling techniques, as detergent washes away the dye, we were unable to simultaneously image their structures. However, our quantitative analysis indicates their proximity, suggesting that the lateral fascicle might play a crucial positional role in MN24 dendritogenesis.

### Loss of Dscam1 disrupts MN24 dendritic processes

**Figure 2.**
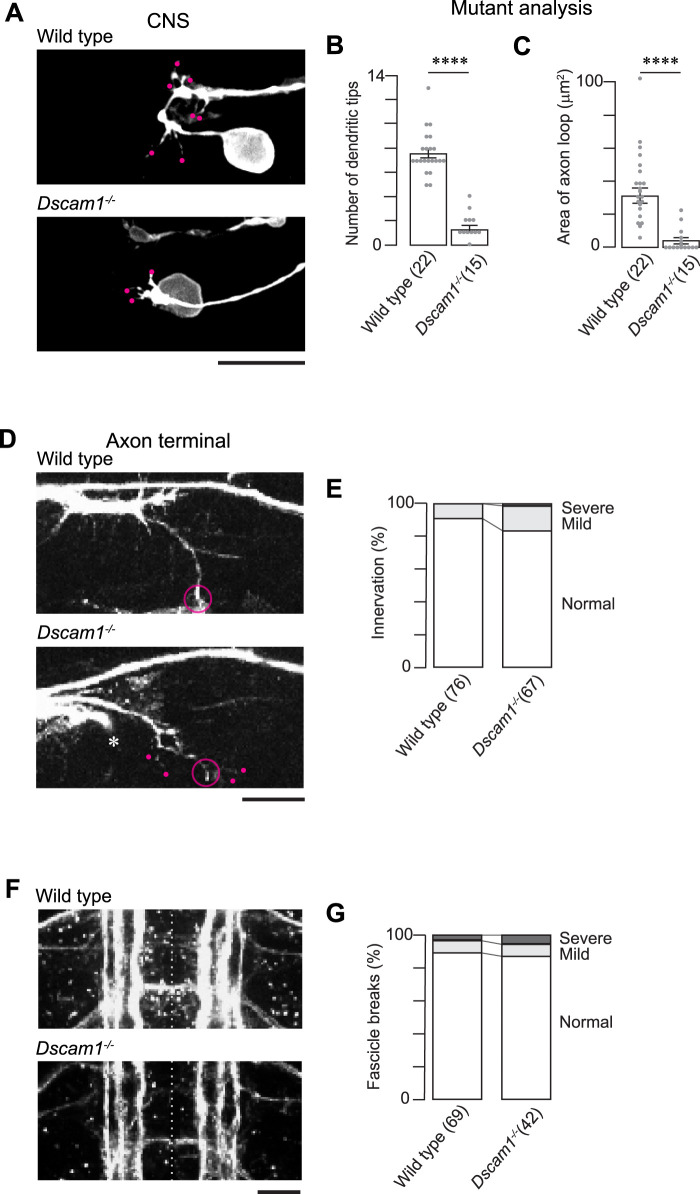
*Dscam1* is required for MN24 neurite development. ***A***, Representative fluorescence images of MN24 within wild-type (top panel) and *Dscam1^−/−^* mutant (bottom panel) backgrounds. ***B***, ***C***, Comparison of mean primary dendritic branch numbers (***B***) and axon routing areas. ***C***, Within wild-type and *Dscam1^−/−^* mutant backgrounds; using Mann–Whitney *U* test. For all graphs, the sample size of neurons is denoted by the number in the parentheses of each genotype unless otherwise specified. For all subsequent statistical analyses, symbols indicate the following: ****p < 0.0001; ***p < 0.001; **p < 0.01; *p < 0.05; ns, not significant. ***D***, Immunofluorescence staining of FasII at 15:00 AEL shows the visual comparison between axon terminals in wild-type (top panel) and *Dscam1^−/−^* mutant (bottom panel) backgrounds. Representative image displaying FasII staining in the wild-type and mutant backgrounds exhibits innervation by the SNa nerve branch (open circle). However, in the *Dscam1^−/−^* mutant background, the SNa sub-branches and the ISNb nerve have some mild targeting defects (magenta and green dots, respectively). ***E***, Quantification of SNa innervation defects within wild-type (*n* = 76 hemisegments) and *Dscam1^−/−^* mutant (*n* = 67 hemisegments) backgrounds. Data is represented as a percentage—number of hemisegments with innervation defects over the total number of hemisegments observed. SNa innervation defects were characterized as mild (light gray) or severe (dark gray) when the SNa sub-branch had targeting defects or the SNa branch did not exit the nerve cord, respectively. ***F***, Representative fluorescence images of FasII-positive axon tracts within wild-type (top panel) and *Dscam1^−/−^* mutant (bottom panel) backgrounds. ***G***, Quantification of lateral fascicle defects within wild-type (*n* = 77 hemisegments) and *Dscam1^−/−^* mutant (*n* = 66 hemisegments) backgrounds. Data is represented as a percentage—length of lateral fascicle defects over total lateral fascicle length. Lateral fascicle defects were characterized as mild or severe when the lateral fascicle was thinning or contained a break, respectively. Scale bars, 10 µm in ***A***, ***D***, ***F***.

As previously demonstrated (Kamiyama et al., 2015), the *Dscam1* gene plays a prominent role in the outgrowth of primary dendritic branches in the aCC motoneuron, evidenced by the near elimination of these branches in the *Dscam1* null mutants (*Dscam1^−/−^*). Building upon this, we investigated whether *Dscam1* similarly regulates dendritogenesis in MN24. We characterized the dendritic outgrowth of MN24 in embryos homozygous for the *Dscam1* mutation. In *Dscam1^−/−^*, we observed a significant decrease in the number of primary dendritic branches. At 15:00 AEL, there were on average 1.3 ± 0.4 dendritic tips in *Dscam1^−/−^* compared with 7.6 ± 0.3 in the wild-type condition (Fig. 2*A*,*B*). This loss-of-dendrite phenotype persisted at a later stage, with *Dscam1^−/−^* having 2.7 ± 0.6 branches compared with 16.0 ± 0.8 in the wild-type condition at 19:00 AEL. Interestingly, in the mutant background, we observed a notable “collapse” in the axonal routing of MN24 (Fig. 2*A*). On average, the area of the axonal routing in the mutants was significantly reduced, measuring only 4.1 ± 4.4 µm^2^, in contrast to the wild-type area, which was 36.2 ± 3.6 µm^2^ (Fig. 2*C*). Further cellular characterization in the *Dscam1^−/−^* mutants revealed that while MN24 extends its axon around the target muscle region in most cases, there were occasional instances where it failed to specifically reach the target muscle 24 (Fig. 2*D–E*). Note that axons of specific motoneurons (e.g., RP1, RP3, RP4, RP5, and some others, which share the ISNb pathway) were more susceptible in the mutant, with 35% of cases exhibiting axon guidance defects. Despite these minor defects, the overall pattern of axon guidance in MN24 remains intact.

Since we hypothesize that the most lateral FasII-positive fascicle might be involved in MN24 dendritogenesis, it is crucial to assess its phenotype in the mutant. Following staining of the *Dscam1^−/−^* mutant with the anti-FasII antibody, we observed thinning in the lateral fascicle and, on some occasions, a “wavy” pattern. However, for the most part, the mutant lateral fascicle appeared relatively normal, where 87.1% of mutant fascicles from the 66 observed hemisegments contained no breakage similar to 89.0% of those from wild-type containing no breakage (Fig. 2*F*,*G*). Based on these observations, we anticipate that the close proximity between this fascicle and MN24 is largely maintained.

In conclusion, our findings indicate that Dscam1 may act as a crucial positional cue for dendritic outgrowth and axonal routing in MN24. This aligns with our previous observations showing a high concentration of Dscam1 proteins at the neuropile, the site of MN24 dendritogenesis, and axonal routing (Kamiyama et al., 2015).

### Developmental time course of MN24 morphogenesis

Next, we sought to assess the development of MN24 to gain insights into the defects in dendritogenesis and axonal routing observed in the mutants. Imaging MN24 at early developmental stages, before its axon reaches the target muscle, precludes the use of retrograde lipophilic dye labeling. Therefore, to characterize the morphological features of MN24, we employed one of the FLP-out techniques (see Materials and Methods for a detailed description; Nern et al., 2015). This method allows for genetic labeling of single *hh-GAL4*-positive neurons through the stochastic expression of a membrane marker. To visualize the fascicles, we used the anti-FasII antibody.

**Figure 3.**
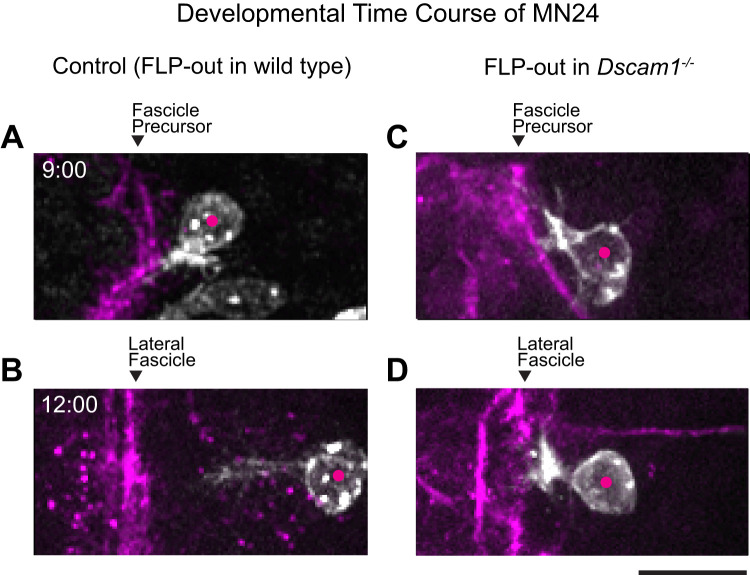
MN24 axon routing supervenes proper cell body localization. ***A****–****D***, Representative fluorescence images of individually labeled MN24 cell bodies within the embryonic CNS in control (***A***, ***B***) and *Dscam1^−/−^* mutant (***C***, ***D***) backgrounds at different developmental stages (9:00 and 12:00 AEL, top and bottom panels, respectively). FLP recombinase-based stochastic labeling was used to genetically label MN24 within the *hh*-GAL4 pattern by expressing a membrane-bound epitope tag (FLAG). Dots represent the position of MN24 soma. Scale bar, 10 µm.

At 9:00 AEL in control embryos, we observed that the cell body of MN24 is localized near the FasII-positive fascicle precursor (Fig. 3*A*). By 12:00 AEL, as the precursor starts to form into distinct fascicles, their position remains the same while the cell body of MN24 moves toward the edge of the VNC. Then, the axon begins to project from the cell body of MN24 (Fig. 3*B*). As shown in Figure 2*A*, the axon makes an acute turn at the intersection with the most lateral fascicle, forming the routing structure by 15:00 AEL. At the site of axonal routing, fine structures appear, indicating the formation of dendritic branches.

In *Dscam1^−/−^* mutants, the cell body localizes at the same position as in control embryos at 9:00 AEL (21.0 ± 1.3 µm for mutant from 10 neurons and 21.4 ± 1.2 µm for control from 11 neurons; *p *= 0.84 using Student’s *t* test; Fig. 3*C*). Notably, it does not move away from the most lateral fascicle and remains in the same position even at 12:00 AEL (Fig. 3*D*). As demonstrated in Figure 2*A*, its axon extends out toward the exit of the VNC with a small or almost no routing pattern by 15:00 AEL; additionally, only a few fine structures are visible at the site where dendrites are supposed to emerge. These results suggest that the loss of *Dscam1* specifically impacts the development of dendrites and axonal routing at different developmental timings. In particular, the routing defect could be attributed to the mislocation of the cell body, which we address in detail in a later section.

### Dual roles of *Dscam1* in dendritic outgrowth and axonal routing in MN24

*Dscam1^−/−^* mutants exhibited two obvious defects in MN24. Because Dscam1 is a cell surface adhesion molecule, which often requires interaction with neighboring cells, the exact mechanism—whether these defects are a direct result of *Dscam1* loss specifically in MN24 or a secondary effect arising from a global loss of *Dscam1*—remains to be elucidated.

**Figure 4.**
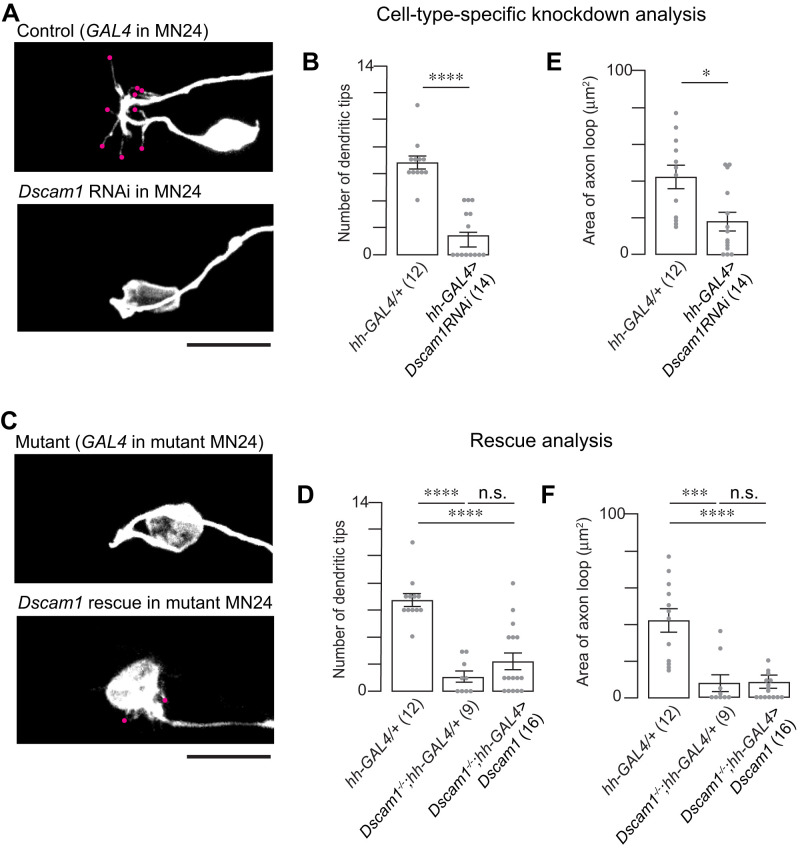
Dscam1 plays a cell-autonomous role for MN24 neurite development. ***A***, Representative fluorescence images of MN24 in wild-type background expressing *hh*-GAL4 driver (top panel) and *Dscam1* RNAi expressed under the control of the *hh*-GAL4 driver (bottom panel). ***B***, ***E***, Comparison of mean primary dendritic branch numbers (***B***) and axon routing areas (***E***) of MN24 in wild-type background expressing *hh-*GAL4 driver and *Dscam1* RNAi expressed under the control of the *hh*-GAL4 driver; using Mann–Whitney *U* test. ***C***, Representative fluorescence images of MN24 in *Dscam1^−/−^* mutant background expressing *hh*-GAL4 driver (top panel) and *Dscam1^−/−^* mutant background resupplied *Dscam1* expressed under the control of the *hh*-GAL4 driver (bottom panel). ***D***, ***F***, Comparison of mean primary dendritic branch numbers (***D***) and axon routing areas (***F***) of MN24 in wild-type background expressing *hh*-GAL4 driver, *Dscam1^−/−^* mutant background expressing *hh*-GAL4 driver, and *Dscam1^−/−^* mutant background resupplied *Dscam1* expressed under the control of the *hh*-GAL4 driver; using Kruskal–Wallis test followed by Dunn’s multiple-comparisons test. Scale bars, 10 µm in ***A***, ***C***.

To further elucidate the mechanism, we conducted cell-type specific manipulation of *Dscam1* using a short hairpin RNA (shRNA) targeting *Dscam1* under *UAS* control for gene knockdown (*UAS- Dscam1 RNAi*). The efficacy of this *UAS- Dscam1 RNAi* line, previously validated (Kamiyama et al., 2015), was apparent when expressed under the control of the pan-neuronal GAL4 driver, *elav-GAL4*, leading to the elimination of Dscam1 proteins from the embryonic CNS. Crossing this RNAi line with *hh-GAL4*, we selectively knocked down *Dscam1* in MN24. This targeted approach resulted in a significant reduction in dendritic branches—on average, MN24-specific RNAi knockdown exhibited only 1.7 ± 0.4 primary dendritic branches, compared with control embryos, which had 6.8 ± 0.5 (Fig. 4*A*,*B*). Notably, reintroducing a single isoform of *Dscam1* (*UAS- Dscam1^exon 17.2^*) into MN24 in the *Dscam1* mutant background did not restore the normal dendritic count (2.3 ± 0.5 for rescue and 1.1 ± 0.7 for mutant control; Fig. 4*C*,*D*).

Regarding axonal routing, the MN24-specific RNAi knockdown of *Dscam1* partially replicated the knockout phenotype. Knocking down *Dscam1* led to alterations in the axonal routing of MN24, with the average routing area measuring 14.0 ± 4.8 µm^2^, compared with the control’s 42.0 ± 5.5 µm^2^ (Fig. 4*A*,*E*). However, this phenotype was less severe than in the knockout control, which had an average loop area of 7.7 ± 5.7 µm^2^ (Fig. 4*F*, second bar). Reintroducing the *Dscam1* gene into MN24 in the *Dscam1^−/−^* background did not significantly rescue the axonal routing structure observed (8.5 ± 4.2 µm^2^ for rescue; Fig. 4*B*,*F*).

From these findings, we draw two conclusions: (1) the RNAi knockdown results suggest that *Dscam1* serves a cell-autonomous function in the dendritogenesis and axonal routing of MN24, and (2) the rescue results indicate that *Dscam1* alone is not sufficient for the formation of both cellular structures in MN24. Furthermore, these results imply the possibility that *Dscam1*, when expressed in other cells, contributes to MN24 morphogenesis, indicating a noncell-autonomous function of Dscam1 in these processes.

### Scp2-GAL4: enabling selective expression of transgenes in lateral fascicles

**Figure 5.**
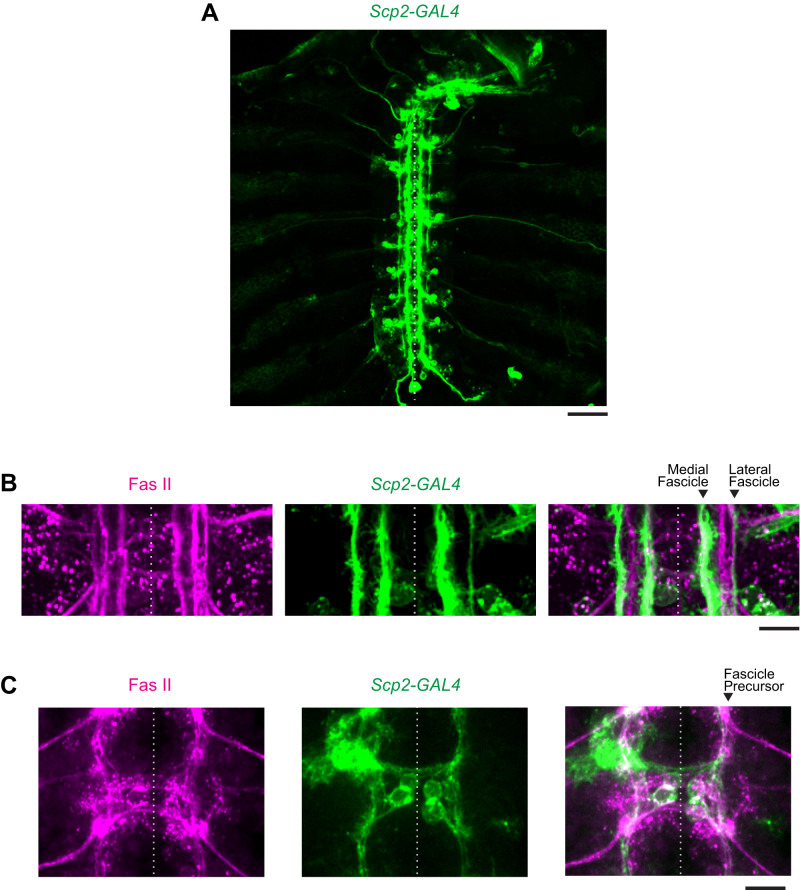
*Scp2*-GAL4 driver allows labeling of lateral fascicle. ***A***, Representative low-magnification images of a 15:00 AEL embryo labeled by membrane-bound GFP under the control of the *Scp2-GAL4* driver. The expression pattern includes longitudinal fascicles but does not label neurons within the SNa nerve tract or afferent sensory axons. ***B***, ***C***, Representative high-magnification images in neuronal fascicles GFP-labeled under the control of *Scp2*-GAL4 driver (green) or immunostained with anti-FasII antibody (magenta). At 15:00 AEL, *Scp2*-positive fascicles include the medial and lateral fascicles (arrowheads) and exclude the intermedial fascicle (***B***). At 9:00 AEL, the *Scp2*-positive expression pattern labels an early fascicle that will later defasciculate in development (***C***). Scale bar, 40 µm in ***A*** and 10 µm in ***B, C***.

To elucidate the noncell-autonomous functions of *Dscam1*, we considered that the most lateral FasII-positive fascicle might provide positional cues to MN24, potentially mediated by Dscam1. To test this hypothesis, we must manipulate the *Dscam1* gene in the lateral fascicle. However, due to the absence of a reported GAL4 line specifically labeling the most lateral fascicle, we embarked on a screening to identify a new GAL4 driver. By crossing ∼20 GAL4 lines with *UAS-mCD4-tdGFP*, we identified a promising candidate, *Scp2-GAL4*. This GAL4 line labels a subset of interneurons that contribute to the formation of the most lateral fascicle. The expression pattern observed in *Scp2-GAL4* highlights neuronal processes from interneurons, segregated into either the medial or lateral fractions of the FasII-positive fascicles (Fig. 5*A*,*B*). Additionally, this GAL4 line targeted aCC and RP2 motoneurons in 40.6 and 34.3% of the observed hemisegments (*n* = 32), respectively. MN3 and MN19 were also labeled, though less frequently, at 6.3% for each of the hemisegments observed. Importantly, *Scp2-GAL4* does not label MN24 or any related motoneurons within the same SNa nerve tract. Furthermore, this GAL4 line does not label any afferent sensory axons within the intersegmental and segmental nerves (Fig. 5*A*). The expression of GFP decorates the FasII-positive fascicle precursor by 9:00 AEL (Fig. 5*C*). In conclusion, we identified *Scp2-GAL4* as a GAL4 line that facilitates the expression of *UAS* transgenes in two of the FasII-positive fascicles at 15:00 AEL, notably including the most lateral fascicle.

### Dscam1 in the lateral fascicle is necessary for MN24 dendritogenesis and axonal routing

**Figure 6.**
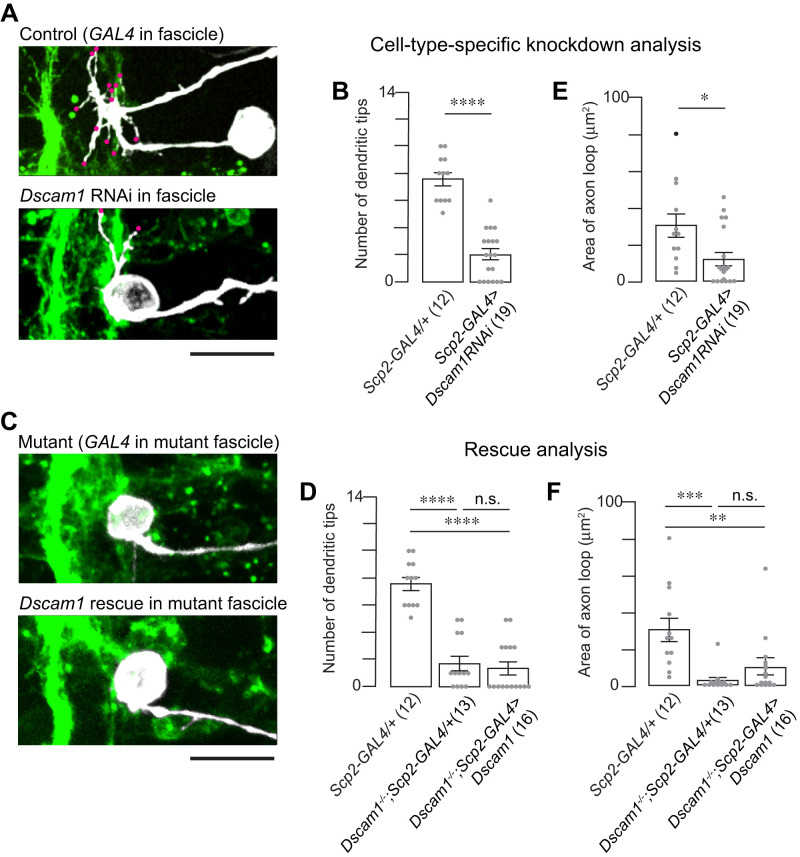
*Scp2*-positive lateral fascicle provides noncell-autonomous Dscam1 for MN24 neurite development. ***A***, Representative fluorescence images of MN24 in wild-type background expressing *Scp2*-GAL4 driver (top panel) and *Dscam1* RNAi expressed under the control of the *Scp2*-GAL4 driver (bottom panel). ***B***, ***E***, Comparison of mean primary dendritic branch numbers (***B***) and axon routing areas (***E***) of MN24 in wild-type background expressing *Scp2*-GAL4 driver and *Dscam1* RNAi expressed under the control of the *Scp2*-GAL4 driver, using Mann–Whitney *U* test. ***C***, Representative fluorescence images of MN24 in *Dscam1^−/−^* mutant background expressing *Scp2*-GAL4 driver (top panel) and *Dscam1^−/−^* mutant background resupplied *Dscam1* expressed under the control of the *Scp2*-GAL4 driver (bottom panel). ***D***, ***F***, Comparison of mean primary dendritic branch numbers (***D***) and axon routing areas (***F***) of MN24 in wild-type background expressing *Scp2*-GAL4 driver, *Dscam1^−/−^* mutant background expressing *Scp2*-GAL4 driver, and *Dscam1^−/−^* mutant background resupplied *Dscam1* expressed under the control of the *Scp2*-GAL4 driver; using Kruskal–Wallis test followed by Dunn’s multiple-comparisons test. Scale bars, 10 µm in ***A***, ***C***. We show lateral fascicles in Dscam1 knockdown and knockout in Extended Data Figure 6-1 and MN24 soma position in Dscam1 knockout in Extended Data Figure 6-2.

Using the *Scp2-GAL4* driver, we simultaneously implemented *UAS-Dscam1 RNAi* for specific gene knockdown and *UAS-mCD4-tdGFP* for targeted cell labeling. This approach led to a significant reduction in dendritic branches—on average, MN24 exhibited 2.0 ± 0.4 primary dendritic branches, compared with the control, which had 8.3 ± 0.6 (Fig. 6*A*,*B*). These results strongly support the concept of a noncell-autonomous function for *Dscam1*. Interestingly, subsequent attempts to rescue the dendritic phenotype by reintroducing *Dscam1* into Scp2-positive neurons were unsuccessful in reversing the mutant phenotype in MN24 (1.4 ± 0.5 for rescue and 2.1 ± 0.5 for mutant control; Fig. 6*C*,*D*). This suggests that the expression of the *Dscam1* gene only in Scp2-positive neurons is not sufficient for MN24 dendritogenesis. It is also important to note that the FasII-positive fascicles, particularly the most lateral fascicle, remain relatively normal despite the knockdown and knockout of *Dscam1* (Extended Data Fig. 6-1).

10.1523/ENEURO.0130-24.2024.f6-1 Figure 6-1 **The most lateral fascicle remains unaffected despite *Dscam1* knockdown and knockout** (**A**-**B**) Representative images of neuronal fascicles GFP-labeled using the *Scp2-GAL4* driver (green) and immunostained with anti-FasII antibody (magenta). Fascicles either coexpressing *Dscam1* RNAi under the control of the same GAL4 driver (**A**) or in a *Dscam1^-/-^* mutant background (**B**) were imaged. FasII-positive medial, intermediate, and lateral fascicles are denoted as M, I, and L. Scale bars, 10 μm. Download Figure 6-1, TIF file.

10.1523/ENEURO.0130-24.2024.f6-2 Figure 6-2 **MN24 Soma Position is Medially Shifted in the *Dscam1^-/-^* Mutant Background** (**A**) Representative images of MN24 at 15:00 AEL in wild-type background expressing *Scp2*-GAL4 driver (green) (top panel) and *Dscam1^-/-^* mutant background expressing *Scp2*-GAL4 driver (bottom panel). Blue and pink bars indicate the distance (μm) from the lateral fascicle and soma, respectively, to the midline. (**B**) Quantification of lateral fascicle position in wild-type background expressing *Scp2*-GAL4 driver and *Dscam1^-/-^* mutant background expressing *Scp2*-GAL4 driver; using Welch’s *t* test. The *Scp2*-positive lateral fascicle does not have a mediolateral shift in the *Dscam1^-/-^* mutant background. (**C**) Quantification of MN24 soma position in wild-type background expressing *Scp2*-GAL4 driver and *Dscam1^-/-^* mutant background expressing *Scp2*-GAL4 driver; using Welch’s *t* test. MN24 soma in the *Dscam1^-/-^* mutant background expressing *Scp2*-GAL4 driver has a more medial shift compared to that of the wild-type background. Scale bar, 10 μm. Download Figure 6-2, TIF file.

Similarly, RNAi knockdown of *Dscam1* using *scp2-GAL4* led to a “collapse” in the axonal routing of MN24. The average axon routing area was measured at 12.6 ± 4.6 µm^2^, significantly reduced compared with the control, which was measured at 35.6 ± 5.8 µm^2^ (Fig. 6*A*,*E*). Additionally, when we resupplied the *Dscam1* gene only to Scp2-positive neurons in *Dscam1^−/−^*, there was no observed rescue of the axonal routing structure (9.4 ± 4.1 µm^2^ for rescue and 2.4 ± 4.0 µm^2^ for mutant control; Fig. 6*B*,*F*). Inspired by the previous observations shown in Figure 3*B* and *D*, the cell bodies of MN24 were differentially positioned relative to the most lateral fascicles in control and *Dscam1^−/−^* embryos: mutant MN24 had a cell body position that seemed more medially shifted compared with control MN24 during development. We measured the positions of both the cell bodies and the fascicle relative to the midline at our observation timepoint, 15:00 AEL. We discovered that while the position of the most lateral fascicle remained unchanged (15.3 ± 0.4 µm for mutant and 14.3 ± 0.5 µm for control), the cell bodies of MN24 were differently positioned, often closer to the lateral fascicle (19.8 ± 0.9 µm for mutant and 26.7 ± 1.0 µm for control; Extended Data Fig. 6-2). This led us to speculate that the reduced area of the axon loop might be a secondary defect—due to the proximity of MN24 cell bodies to the most lateral fascicle, there may be insufficient space for the axonal routing to form properly in these genetic backgrounds (see Discussion).

### Dscam1 mediates interaction between MN24 and the lateral fascicle for proper dendritogenesis and axonal routing of MN24

**Figure 7.**
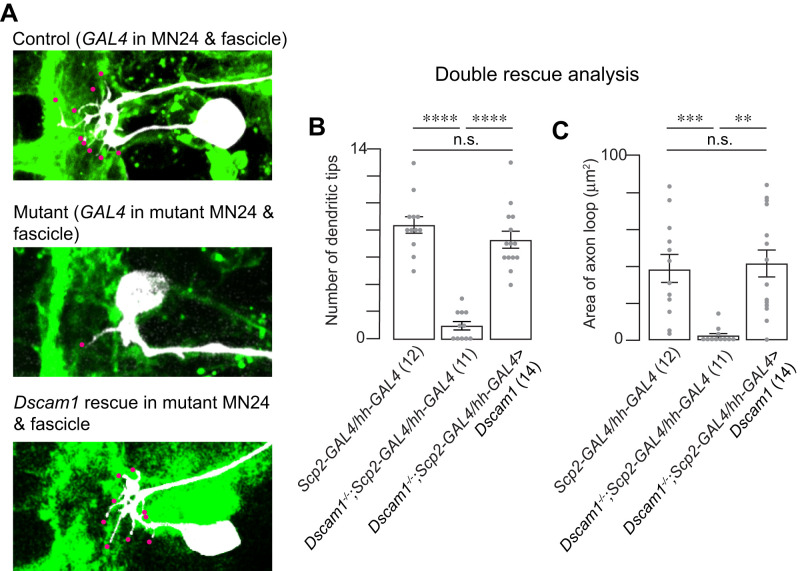
Dscam1 in both Scp2-positive lateral fascicle and MN24 is sufficient to restore MN24 dendritogenesis and axon routing. ***A***, Representative fluorescence images of MN24 within wild-type background (top panel), *Dscam1^−/−^* mutant background with combined *Scp2*- and *MN24*-specific expression of membrane-bound GFP (middle panel), and *Dscam1^−/−^* mutant background with combined *Scp2*- and *MN24*-specific resupply of *Dscam1* (bottom panel). Scale bar, 10 µm. ***B***, ***C***, Comparison of mean primary dendritic branch numbers (***B***) and axon routing area (***C***) among MN24 in wild-type background, *Dscam1^−/−^* mutant background with *Scp2*- and *MN24*-specific expression of GFP membrane-bound, and *Dscam1^−/−^* mutant background with combined *Scp2*- and *MN24*-specific resupply of *Dscam1*, using Kruskal–Wallis test followed by Dunn’s multiple-comparisons test.

**Figure 8.**
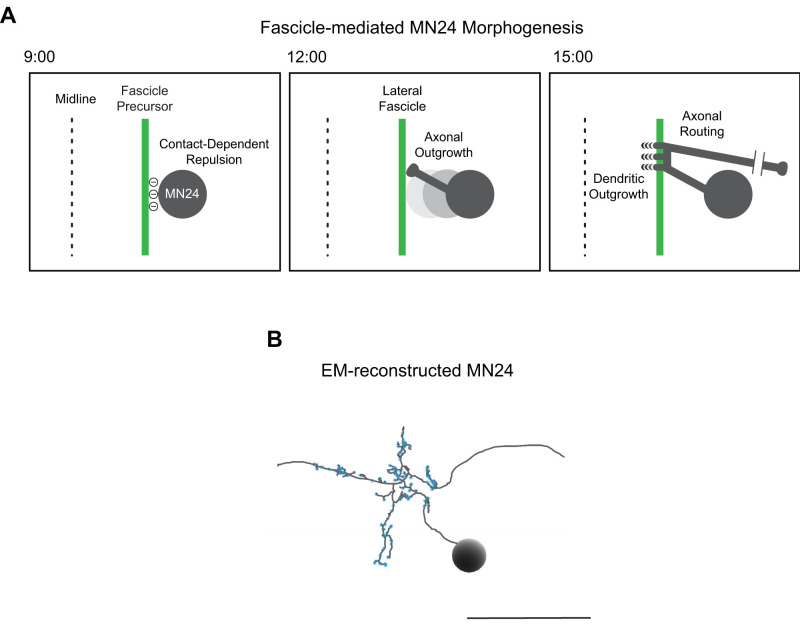
Proposed model for fascicle-mediated MN24 morphogenesis. ***A***, Schematic illustrating the proposed model of how the fascicle structure mediates MN24 dendrite outgrowth and soma localization. ***B***, Electron microscopy (EM) reconstruction of a single MN23/24 in first instar larva. Prominent morphological structures such as dendritic outgrowth and axon routing are retained in larval MN24. The backbone is indicated by gray. Blue dots indicate synaptic sites. Scale bar, 20 µm.

Our experiments indicate that both cell-autonomous and noncell-autonomous functions of *Dscam1* are essential for dendritogenesis and axonal routing in MN24 and suggest that Dscam1 on either side of the neuronal membranes function to guide MN24 neurite processes. If Dscam1 serves as a positional cue, then we reasoned that providing Dscam1 to both MN24 and Scp2-positive neurons would restore the MN24 mutant phenotype. To directly test this hypothesis, we reintroduced *UAS-Dscam1* into *Dscam1^−/−^* mutants using two GAL4 drivers, *Scp2-* and *hh-GAL4*, targeting both Scp2-positive fascicle and MN24. In alignment with our hypothesis, this dual reintroduction of *Dscam1* led to a complete recovery of the MN24 dendrite count (7.4 ± 0.5 for rescue and 8.4 ± 0.6 for control) and restoration of the axonal routing structure (41.1 ± 6.2 µm^2^ for rescue and 38.6 ± 6.7 µm^2^ for control; Fig. 7*A–C*). Notably, the axonal structure recovered, with the cell bodies repositioning to locations similar to those in the controls (27.1 ± 0.9 µm for rescue and 28.6 ± 1.0 µm for control; Extended Data Fig. 7-1). These findings suggest that Dscam1’s function in both MN24 and Scp2-positive neurons is crucial for dendritogenesis and axonal routing in MN24 and are consistent within the model that Dscam1 acts as a positional cue to guide MN24 development (Fig. 8*A*).

10.1523/ENEURO.0130-24.2024.f7-1 Figure 7-1 **Resupplying *Dscam1* in Scp2-Positive Lateral Fascicle and MN24 Restores Mutant MN24 Soma Position** Quantification of MN24 soma positions in wild-type background with *Scp2*- and *hh*-specific expression of membrane-bound GFP, *Dscam1^-/-^* mutant background with *Scp2*- and *hh*-specific expression of membrane-bound GFP, and *Dscam1^-/-^* mutant background with combined *Scp2*- and *hh*-specific resupply of *Dscam1*; using Kruskal–Wallis test followed by Dunn’s multiple comparisons test. Download Figure 7-1, TIF file.

## Discussion

### Dscam1 as a positional cue defines the MN24 dendritogenesis site

Expanding on our previous findings about dendritogenesis in the aCC motoneuron (Kamiyama et al., 2015), our current study explores the function of *Dscam1* during MN24 dendritogenesis. Our research presents several lines of evidence suggesting that the interaction between MN24 and Scp2-positive neurons is critical for the outgrowth of primary dendritic branches in MN24. Firstly, we demonstrate that MN24 and the Scp2-positive fascicle are in proximity. Secondly, upon knocking down *Dscam1* in either MN24 or Scp2-positive fascicle, we observed a reduction in the number of primary dendritic branches, mirroring the phenotype seen in *Dscam1^−/−^* mutants. This suggests that *Dscam1* in both MN24 and Scp2-positive fascicle is necessary for MN24 dendritogenesis. Thirdly, our rescue experiments in *Dscam1^−/−^* mutants, involving the reintroduction of *Dscam1* into either MN24 or Scp2-positive fascicle, were unsuccessful. This indicates that *Dscam1* function in either neuron type alone is insufficient. However, when *Dscam1* was reintroduced into both MN24 and Scp2-positive fascicle in *Dscam1^−/−^* mutants, we could fully rescue the dendritic phenotype. Finally, the observation that MN24 axons still contact the Scp2-positive fascicle in *Dscam1^−/−^* mutants rules out the possibility that the reduced number of dendrites is due to a mislocation of these neural processes. Consequently, we propose that Dscam1 provides a positional cue for MN24 through cell–cell contact, defining the site of dendritic outgrowth (Fig. 8*A*). This mechanism echoes how vertebrate DSCAM guides retinal ganglion cell (RGC) dendrites and bipolar cell axons for synapse formation in the chick retina (Yamagata and Sanes, 2008), suggesting a potentially conserved principle in dendritic outgrowth mediated by interneuronal Dscam1 interactions.

Our study specifically targets the early developmental stage of the MN24, around 15:00 after egg laying (AEL), a pivotal time when primary dendritic branches start to emerge. The critical role of these initial branches in forming the foundation for higher-order branches and synaptic formations has yet to be fully established, but emerging data offer promising insights. Recent advancements in comprehensive connectome efforts have facilitated the reconstruction of the entire CNS in the first instar larva, and this valuable data is accessible in a publicly available database (Saalfeld et al., 2009; Winding et al., 2023). Using this resource, we have examined an electron microscopy (EM) reconstructed model of MN24. This model revealed that the higher-order branches are situated in the regions of the axonal turning points, corresponding to the area where MN24’s primary dendrites initiate (Fig. 8*B*). Notably, these branches exhibit numerous synapses throughout their structures. This observation suggests a developmental progression from primary dendritic branches to the establishment of functional synapses in MN24.

### The roles of Dscam1 in axonal routing and soma mislocation of MN24

In addition to the observed loss-of-dendrite phenotype, our study has revealed axonal routing defects in MN24 in *Dscam1^−/−^* mutants. Normally, MN24 axons project ventrally, reaching the most lateral FasII-positive fascicle and then undergo a crucial lateral turn as part of their axonal routing process. However, in *Dscam1^−/−^* mutants, this axonal routing is notably compromised. One potential explanation for this diminished axonal routing in *Dscam1^−/−^* mutants could be related to a mislocation of the soma in MN24. In *Dscam1^−/−^* mutants, the soma position is observed to be closer to the lateral fascicle (Figs. 6*C*, 7*A*; Extended Data Fig. 6-2). Interestingly, reintroducing *Dscam1* into both MN24 and Scp2-positive neurons corrects the soma’s position, in addition to the restoration of the normal axonal loop structure (Fig. 7*A*, Extended Data Fig. 7-1).

The role of *Dscam1* in soma migration during brain development in *Drosophila* is an emerging research interest. A critical study by Liu et al. focusing on larval medulla neurons has provided significant insights into this process (Liu et al., 2020). They revealed that within the fly visual system, the cell bodies of sister neurons from the same lineage exhibit mutual repulsion. This event contributes to the formation of columnar structures. This repulsive interaction is mediated by interneuronal interaction between Dscam1. Inspired by these findings, we propose a similar mechanism in the MN24 system. We propose a model where Dscam1 orchestrates a repulsive interaction between the soma of MN24 and the Scp2-positive fascicle (Fig. 8*A*). Dscam1 might play an initial permissive role, where the interneuronal Dscam1 interaction mediates the repulsion between the early fascicle structure and MN24 soma at 9:00 AEL. Once the MN24 soma moves away from the lateral fascicle by 12:00 AEL, other molecular players—likely derived from the lateral fascicle—may guide and form the axon routing structure in a Dscam1-independent manner. This Dscam1 independency is supported by our observations that, on some occasions, *Dscam1^−/−^* mutants are still capable of forming routing structures (Fig. 4*C*, mutant MN24). Due to the limited space between MN24 and the most lateral fascicle, however, the routing structures are very small or often undetectable.

### Single isoform of Dscam1 for rescue in morphological defects in MN24

An impressive diversity of 19,008 isoforms, each with different extracellular domains, can arise from the *Dscam1* gene through alternative splicing of three variable exon clusters (Schmucker et al., 2000; Tomer et al., 2012; Sun et al., 2013). These extracellular domains can bind in a homophilic and isoform-specific manner (Wojtowicz et al., 2004, 2007). Intriguingly, each neuron in the fly is found to express a distinct and limited set of Dscam1 isoforms (Hattori et al., 2007, 2009). Consequently, the isoform-specific binding characteristics of Dscam1 facilitate homophilic repulsion exclusively among identical (or “self”) cells, raising questions about Dscam1 interactions between different neuron types like MN24 and Scp2-positive neurons.

In our experiments, we introduced a single isoform of *Dscam1* simultaneously into different neuron types, which successfully rescued the phenotypes associated with dendritogenesis and axonal routing in MN24 (Fig. 7). Our finding echoes a previous result showing that a single isoform of *Dscam1* can rescue an axon scaffold positive for anti-BP102 in the embryonic CNS (Hernandez et al., 2023), suggesting that just one isoform of *Dscam1* is sufficient for many developmental processes. This leads us to question the nature of Dscam1 trans- and homophilic interactions between different neuronal types. Several hypotheses arise: one possibility is that MN24 and Scp2-positive neurons express the same set of isoforms, potentially due to originating from the same neuronal progenitor cells, thus sharing isoform profiles. Alternatively, the trans-interaction of Dscam1 might be mediated by other molecules, forming a protein complex. For instance, in *Caenorhabditis elegans*, the dendritic branching of PVD neurons involves the interaction of SAX7/NMR-1 transmembrane proteins with DMA-1, mediated by the secreted LECT-2 adapter (Zou et al., 2016; Sundararajan et al., 2019). A similar mechanism might be at play in *Drosophila*, with secreted molecules [such as Slit (Dascenco et al., 2015; Alavi et al., 2016), Netrin (Andrews et al., 2008; Liu et al., 2009), or other ligands yet to be determined] bridging opposing Dscam1 membranes through their nonvariable regions.

### Cross-species insights into DSCAM-mediated motor circuit formation

Unraveling the specific mechanisms of Dscam1 interactions among diverse neuronal types will significantly broaden our understanding of how our model generalizes to motoneurons in *Drosophila*. Additionally, the structural and functional similarities between the *Drosophila* embryonic CNS and the mammalian spinal cord highlight the potential for cross-species studies on DSCAM. The spinal cord, within the neural tube, serves as a model for axon guidance research, showcasing shared molecular mechanisms between mammals and *Drosophila* (Evans and Bashaw, 2010; Evans, 2016; Howard et al., 2019). For instance, the interaction between Netrin1 and DCC, which directs commissural axons toward the midline in mice, reflects analogous processes in the *Drosophila* embryonic CNS (Harris et al., 1996; Kolodziej et al., 1996; Mitchell et al., 1996; Fazeli et al., 1997). Recent findings from Klar’s group have significantly emphasized the role of homophilic DSCAM interactions in the fasciculation of chick commissural axons (Cohen et al., 2017). Their in situ hybridization data reveal that *DSCAM* is expressed in subsets of motoneurons. Considering the close proximity of motoneuron cell bodies and dendrites to these commissural axons (Avraham et al., 2009, 2010), it is plausible that axonal fascicles could influence motoneuron morphogenesis through DSCAM-mediated interactions. Future research along these lines is essential.

## Synthesis

Reviewing Editor: Fabienne Poulain, University of South Carolina Decisions are customarily a result of the Reviewing Editor and the peer reviewers coming together and discussing their recommendations until a consensus is reached. When revisions are invited, a fact-based synthesis statement explaining their decision and outlining what is needed to prepare a revision will be listed below. The following reviewer(s) agreed to reveal their identity: NONE. Note: If this manuscript was transferred from JNeurosci and a decision was made to accept the manuscript without peer review, a brief statement to this effect will instead be what is listed below.

Thank you for submitting your manuscript to eNeuro. After careful consideration, the reviewers and I agree that your study investigating the functions of DSCAM in motorneuron soma positioning, axonal routing and dendritic development in vivo is interesting and significant. In particular, the role uncovered for DSCAM in mediating inter-neuronal communication independently of its isoform diversity represents an advancement in the field. However, reviewers also agree that several points detailed below, and in particular the interaction between fascicle and MN24 neuron, need to be addressed before your manuscript can be considered for publication.

Major points:

1) The model suggests that the MN24 projection reaches the lateral fascicle and neuron-neuron interactions via Dscam1 mediate the sharp turn of the axon. However, it remains unclear whether Dscam1 is directly guiding the axon, or the axon is captured as the soma migrates away from the midline depending on when cell-cell contact is first observed. The authors can test for cell-cell interactions between neurons in the fascicle and MN24 with GRASP. A hh-LexA is available (Wendler et al., 2022 Scientific Reports) and can be used with the Scpr2-GAL4 to drive the corresponding GRASP components.

2) Is there any possibility that the dscam1 mutant phenotype is due to a delay in development of MN24? What is the morphology and trajectory of the MN24 projection at earlier stages? Also, does the morphology and cell body position in later developmental stages (e.g. L1-3) recover or remain altered?

3) In Figure 7C, there is a full fascicle in each of the images, but are those the lateral-most fascicles or the medial-lateral fascicles? If the latter, then what happened to the lateral fascicle? And if the lateral fascicle is not present, then how does that contribute to their model?

4) In Figure 3D, in the dscam1 mutant, the ISNb branch is not present. Is that nerve defective with loss of dscam1, similar to ISNb failure to innervate muscles 6/7 in dscam1 mutants (Alavi et al., 2016)? Or could this also be due to a developmental delay?

Minor points:

1) The authors state that "Scp2-GAL4 does not label MN24 or any related motoneurons within the same SNa nerve tract." However, are there any afferent sensory axons within the SN nerve? If so, could those contribute? Also, the different nerve tracts eventually meet in a larger nerve. Is the expression of Scp2-GAL4 a concern? Additionally, a zoomed out image of the VNC and body wall should be included in the supplemental section to show the expression pattern.

2) Figure 7E, F: The y-axis is the "Square area of axon loop". What is the "square" here? Some similar graphs state "square" others leave out the "square".

3) Figures 1 and 2 could be combined as they both describe wildtype parameters.

4) The Dscam1 gene has a capital D in Flybase, even though it is recessive because it was named after a homologue first identified in humans.

5) Please indicate stages in figure legends.

6) In Figure 3, label 3A as CNS (reasonably obvious with cell body visible) and 3D with axon terminals/SNa for clarity.

7) Previous work (K. Hernandez et al., 2023, Dev. Dyn.) has demonstrated single Dscam1 isoform rescue in the CNS axon scaffold.

## Author Response

Synthesis of Reviews:

Synthesis Statement for Author (Required):

Thank you for submitting your manuscript to eNeuro. After careful consideration, the reviewers and I agree that your study investigating the functions of DSCAM in motorneuron soma positioning, axonal routing and dendritic development in vivo is interesting and significant. In particular, the role uncovered for DSCAM in mediating inter-neuronal communication independently of its isoform diversity represents an advancement in the field. However, reviewers also agree that several points detailed below, and in particular the interaction between fascicle and MN24 neuron, need to be addressed before your manuscript can be considered for publication.

We thank the reviewers for their constructive and positive comments and appreciate their recognition of the significance of our work on Dscam1 in inter-neuronal communication during motoneuron development. We have addressed all the comments raised in the Synthesis Statement, and we believe these revisions enhance the manuscript's logical clarity and finally present a stronger model.

We placed considerable focus on addressing Major Points 1 and 2. The primary concerns raised were: (1) the timing of contact between the MN24 projection and the lateral fascicle, and (2) the mechanisms of Dscam1-mediated morphogenesis during development. To address these issues, we analyzed MN24 development in both wild-type and Dscam1 mutant backgrounds. This analysis allowed us to define critical developmental time points when MN24 and the lateral fascicle interact. Furthermore, our genetic analysis of Dscam1 mutants suggests a role for Dscam1 in dendritogenesis and axonal routing in MN24, as detailed below.

Additionally, we have included more descriptions and images that were previously omitted, addressing the critiques raised in Major Points 3 and 4, as well as minor concerns. Some figures have been rearranged and split into Extended Data Figures, following the reviewers' suggestions. Changes in the text are highlighted in red for clarity. We hope these revisions will lead to a positive response and acceptance for publication.

Major points:

1) The model suggests that the MN24 projection reaches the lateral fascicle and neuron-neuron interactions via Dscam1 mediate the sharp turn of the axon. However, it remains unclear whether Dscam1 is directly guiding the axon, or the axon is captured as the soma migrates away from the midline depending on when cell-cell contact is first observed. The authors can test for cell-cell interactions between neurons in the fascicle and MN24 with GRASP. A hh-LexA is available (Wendler et al., 2022 Scientific Reports) and can be used with the Scpr2-GAL4 to drive the corresponding GRASP components.

We thank the reviewers for the opportunity to reevaluate our model. After conducting the suggested experiments, as described in Major Points 1 and 2, which examined the initial interactions between the lateral fascicle and MN24, we have refined our model.

We summarized our efforts based on the reviewers' suggestions as follows:

Initially, we planned to use the GRASP approach; however, the hh-LexA line was unavailable from any stock center. We reached out to both corresponding authors from Wendler et al., 2022 Scientific Reports. Unfortunately, one author did not have the line, and the other was in the process of relocating their lab to another country, making it inaccessible for our experiments in time.

As a result, we turned to the available Scp2-LexA lines. We characterized the expression patterns within the lateral fascicle of five Scp2-LexA lines from the Bloomington Drosophila Stock Center. Upon analysis, we found that the expression patterns of Scp2-GAL4 and Scp2- LexA differed. Specifically, expression in the lateral fascicle was observed in only two of these lines. However, these two lines also showed broad expression in MN24 and other related motoneurons within the SNa nerve tract, indicating that they would not be compatible for our GRASP experiment.

Based on these results, we turned to another approach to assess the interactions. We employed the FLP-out technique, specifically using the MultiColor FLPOut (MCFO) system.

The primary reason for selecting this system was its inclusion of a membrane marker with spaghetti monster GFP. The technical details of this method are carefully described in the Materials and Methods section on page 23, lines 453 to 466. This system, used in conjunction with hh-GAL4, enabled us to visualize the development of MN24 processes at single-cell resolution. To visualize the fascicle structure, we used the anti-FasII antibody.

In the new Figure 3A-B, we present images of MN24 at different time points. At 9:00 AEL in control embryos, the cell body of MN24 is localized in close proximity to the FasII-positive fascicle precursor. By 12:00 AEL, the cell body has moved away from the fascicles, and the axon begins to project from the cell body. By 15:00 AEL, the axon routing structure is forming, making an acute turn at the intersection with the lateral fascicle. These results suggest that soma positioning occurs prior to the development of the axon routing structure in MN24. We describe this observation on the page 10, lines 167 to 182.

This observation contradicts the latter model, which suggested that "the axon is captured as the soma migrates away from the midline." This discrepancy motivated us to assess MN24 development in Dscam1-/- mutants (see Major Point 2).

2) Is there any possibility that the dscam1 mutant phenotype is due to a delay in development of MN24? What is the morphology and trajectory of the MN24 projection at earlier stages? Also, does the morphology and cell body position in later developmental stages (e.g. L1-3) recover or remain altered? This critique is related to Major Point 1. We believed that comparing MN24 development in control vs. Dscam1-/- mutants would help determine whether the observed phenotype is due to a delay in development or not.

In our response to Major Point 1, we examined the morphology of MN24 at earlier stages in control embryos and found that by 12:00 AEL, the cell body of MN24 is already positioned away from the fascicle structure. However, in Dscam1-/- mutants, the cell body remains close to the fascicle from 12:00 AEL onward (this new observation is also stated in the main text on pages 10 to 11, lines 183 to 192 as well as Figure 3C-D). If our observations were due to a delay in the development of MN24, we would expect to see the cell body move away from the fascicle by 15:00 AEL. However, this was not the case, suggesting that the phenotype is not merely a developmental delay but rather a specific effect of the Dscam1 mutation on MN24 morphogenesis.

As suggested in Major Point 2, we also assessed the dendritic morphology and cell body position at later developmental stages. Because the Dscam1-/- mutant is embryonic lethal before reaching the L1 stage, we examined MN24 in the embryonic stage just before larval hatching. Compared to wild-type MN24 (which have 16.0 {plus minus} 0.8 dendritic tips), the mutant MN24 have significantly fewer dendrites (2.7 {plus minus} 0.6) at 18:00 AEL, suggesting that dendritic formation remains largely disrupted even in later development (this is stated on pages 8 to 9, lines 141 to 145). Additionally, we did not observe noticeable changes in cell body position. Altogether, these findings suggest that the observed phenotypes, such as dendritic and soma mislocation defects, are unlikely to be due to a delay in MN24 development.

Based on the results from Major Points 1 and 2, we refined our model as follows: At 9:00 AEL, the early fascicle structure and MN24 interact through Dscam1-dependent contact, leading to a repulsion that allows them to separate. By 12:00 AEL, MN24 and the lateral fascicle are properly localized, and MN24 begins to extend and guide its axon, forming an axon routing structure. By 15:00 AEL, at the intersection between the MN24 axon and the lateral fascicle, MN24 initiates dendritic outgrowth via Dscam1-Dscam1 interaction.

It remains unclear whether this axonal routing is directly mediated by Dscam1 or involves other molecular players. We believe other factors derived from the lateral fascicle are involved, as Dscam1-/- mutants often exhibit routing structures, although these structures are notably small due to the limited space between MN24 and the lateral fascicle.

Our new model is stated on page 18, lines 343 to 351. Additionally, we have updated the drawing of our model as shown in Figure 8A, to reflect these insights.

3) In Figure 7C, there is a full fascicle in each of the images, but are those the lateral-most fascicles or the medial-lateral fascicles? If the latter, then what happened to the lateral fascicle? And if the lateral fascicle is not present, then how does that contribute to their model? Thank you for bringing this to our attention, and we apologize for any confusion caused. We should have included images of co-immunostaining for GFP and FasII to clearly show the lateral fascicles. In Figure 6C (corresponding to the previous Figure 7C), we represented only the GFP signal with MN24 using a lipophilic dye. This was due to the incompatibility of our dye approach with immunofluorescence, preventing us from showing FasII-positive fascicles.

To address this, we have now included images of co-immunostaining for GFP and FasII in Dscam1 knockdown and knockout embryos in the new Extended Data Figure 6-1. FasII staining clearly reveals the presence of three fascicles even in mutant embryos, with the lateral and medial fascicles being positive, but not the intermediate ("medial-lateral") fascicle. The fascicle in contact with MN24 is indeed the most lateral fascicle. In addition to the new figures, we have included an explanation of this observation on page 13, lines 251 to 254.

4) In Figure 3D, in the dscam1 mutant, the ISNb branch is not present. Is that nerve defective with loss of dscam1, similar to ISNb failure to innervate muscles 6/7 in dscam1 mutants (Alavi et al., 2016)? Or could this also be due to a developmental delay? We thank the reviewer for highlighting this observation. We carefully examined the ISNb nerve in Dscam1-/- mutants at 15:00 AEL and found that approximately 65% of the ISNb branch was able to project normally to the peripheral muscles. However, we observed some targeting defects in the ISNb branch (10% mild, 25% severe). To determine whether the ISNb branch is defective or simply developmentally delayed, we examined a later time point: at 18:00 AEL, similar to the developmental stage where Alavi et al. observed some ISNb innervation defects (specifically, 16% muscle 6/7 innervation defects). We observed targeting defects in the ISNb innervation (31% mild, 29% severe). Some differences in observed ISNb defects could be due to the different Dscam1 mutants employed between our group (amorphic allele; Dscam121) and Alavi et al. (hypomorphic allele; Dscam1P). Overall, these observations suggest that the ISNb nerve is defective with the loss of Dscam1, as opposed to being developmentally delayed.

Because our focus has been on the SNa nerve, our description was biased toward the branching pattern of SNa. To provide at least minimum information on the ISNb nerve, we have included the description of ISNb defects in the main text (page 9, lines 151 to 153) and updated the figure legend of Figure 2D (page 32, lines 688 to 690).

Minor points:

1) The authors state that "Scp2-GAL4 does not label MN24 or any related motoneurons within the same SNa nerve tract." However, are there any afferent sensory axons within the SN nerve? If so, could those contribute? Also, the different nerve tracts eventually meet in a larger nerve. Is the expression of Scp2-GAL4 a concern? Additionally, a zoomed out image of the VNC and body wall should be included in the supplemental section to show the expression pattern.

We thank the reviewer for this suggestion. To address the expression pattern of the Scp2- GAL4 driver, we provide a zoomed-out image of the embryo fillet preparation, showing that the Scp2-GAL4 expression is primarily in the VNC. There is little to no expression in the peripheral body wall or afferent sensory axons, which alleviates concerns that the Scp2-GAL4 may drive non-fascicle-specific effects on MN24 development. We added this in the new Figure 5A and in the main text on pages 12 to 13, lines 235 to 238.

2) Figure 7E, F: The y-axis is the "Square area of axon loop". What is the "square" here? Some similar graphs state "square" others leave out the "square".

Thank you for bringing this to our attention. We have updated the figures to remove "square" and now state simply "area of axon loop" in all figures.

3) Figures 1 and 2 could be combined as they both describe wildtype parameters.

Thank you for this suggestion; we have set Figure 2 as Extended Data Figure 1-1. We also rearranged several figures and moved three figures into Extended Data Figures.

4) The Dscam1 gene has a capital D in Flybase, even though it is recessive because it was named after a homologue first identified in humans.

We changed the text and figures to reflect this suggestion.

5) Please indicate stages in figure legends.

We updated our Figure 1 legend to indicate the developmental stage, stating that "all subsequent images are taken at 15:00 AEL unless otherwise specified." 6) In Figure 3, label 3A as CNS (reasonably obvious with cell body visible) and 3D with axon terminals/SNa for clarity.

We agree with the reviewer and have updated our figure to reflect this clarification in Figure 2A and D (corresponding to the previous Figure 3A and D).

7) Previous work (K. Hernandez et al., 2023, Dev. Dyn.) has demonstrated single Dscam1 isoform rescue in the CNS axon scaffold.

We thank the reviewer for pointing out this recent work, which we have now included in the statement on page 19, lines 364 to 367 of our main text.

Besides these changes, we also included new information on Fly Stock and Immunohistochemistry for the FLP-out assay using MCFO in the Materials and Methods.
