# Supplementary figures and images for: Adjacent Neuronal Fascicle Guides Motoneuron 24 Dendritic Branching and Axonal Routing Decisions through Dscam1 Signaling
Source: eNeuro. 2024 Oct 18;11(10):ENEURO.0130-24.2024. doi: 10.1523/ENEURO.0130-24.2024 (PMC11495862; doi:10.1523/ENEURO.0130-24.2024)

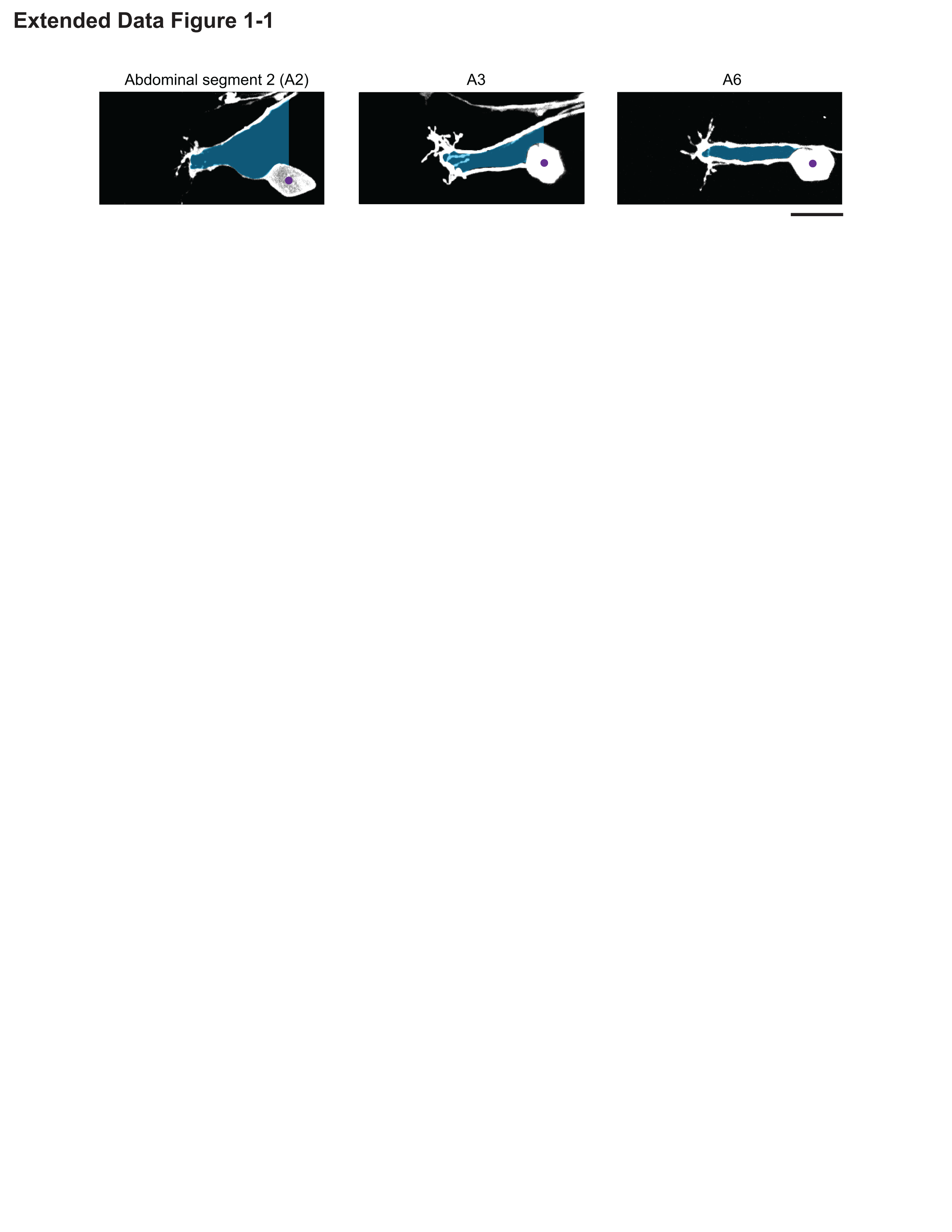

Supplement: Figure 1-1 — Segment-specific MN24 morphologies in the Wild-Type Background Representative images depicting the morphology of wild-type MN24 in different abdominal segments are shown. These images show the characteristic dendrites and axon routing, observed in Figure 1A. Notably, the angle of the axon segment projecting towards the muscle varies in a segment-specific manner. Axon routing area (shaded blue) is measured as the area within the loop. For “open” axon routing areas, (left and middle panels), we define the center of the cell body (purple dot) and use the perpendicular line to the soma center as the border for measurement of the axon routing area. Scale bar, 10 μm. Download Figure 1-1, TIF file. [file eneuro-11-ENEURO.0130-24.2024-s002.tif]

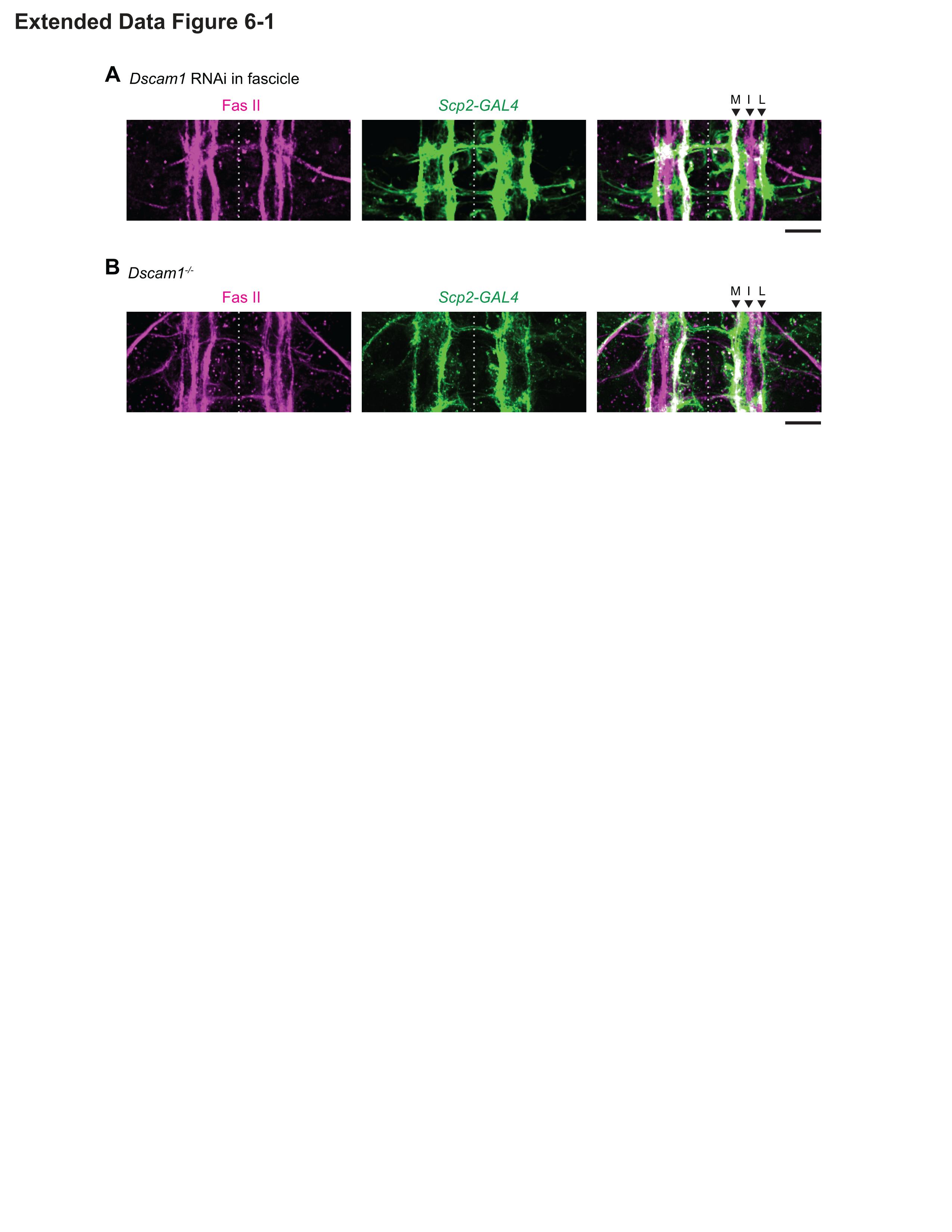

Supplement: Figure 6-1 — The most lateral fascicle remains unaffected despite Dscam1 knockdown and knockout (A-B) Representative images of neuronal fascicles GFP-labeled using the Scp2-GAL4 driver (green) and immunostained with anti-FasII antibody (magenta). Fascicles either coexpressing Dscam1 RNAi under the control of the same GAL4 driver (A) or in a Dscam1-/- mutant background (B) were imaged. FasII-positive medial, intermediate, and lateral fascicles are denoted as M, I, and L. Scale bars, 10 μm. Download Figure 6-1, TIF file. [file eneuro-11-ENEURO.0130-24.2024-s003.tif]

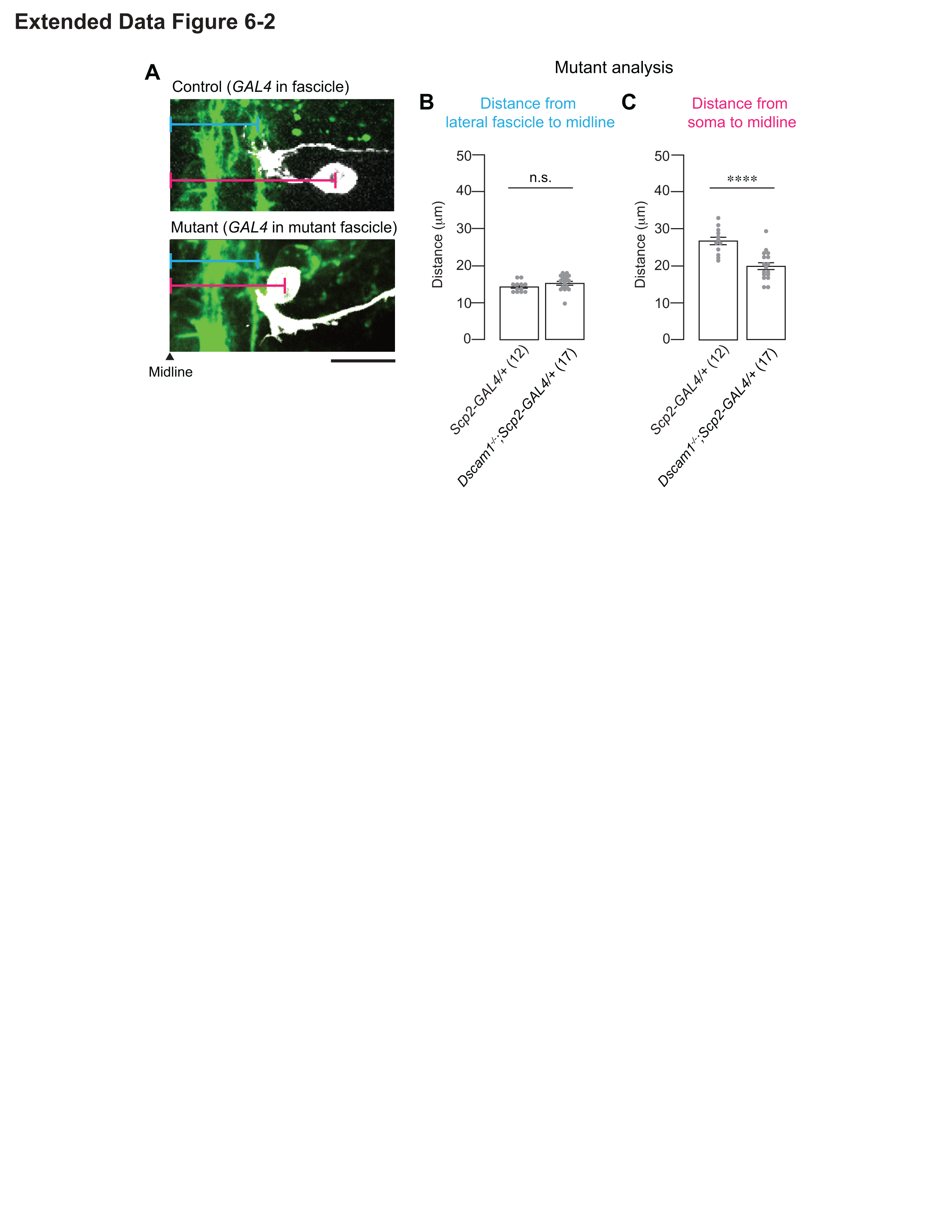

Supplement: Figure 6-2 — MN24 Soma Position is Medially Shifted in the Dscam1-/- Mutant Background (A) Representative images of MN24 at 15:00 AEL in wild-type background expressing Scp2-GAL4 driver (green) (top panel) and Dscam1-/- mutant background expressing Scp2-GAL4 driver (bottom panel). Blue and pink bars indicate the distance (μm) from the lateral fascicle and soma, respectively, to the midline. (B) Quantification of lateral fascicle position in wild-type background expressing Scp2-GAL4 driver and Dscam1-/- mutant background expressing Scp2-GAL4 driver; using Welch’s t test. The Scp2-positive lateral fascicle does not have a mediolateral shift in the Dscam1-/- mutant background. (C) Quantification of MN24 soma position in wild-type background expressing Scp2-GAL4 driver and Dscam1-/- mutant background expressing Scp2-GAL4 driver; using Welch’s t test. MN24 soma in the Dscam1-/- mutant background expressing Scp2-GAL4 driver has a more medial shift compared to that of the wild-type background. Scale bar, 10 μm. Download Figure 6-2, TIF file. [file eneuro-11-ENEURO.0130-24.2024-s004.tif]

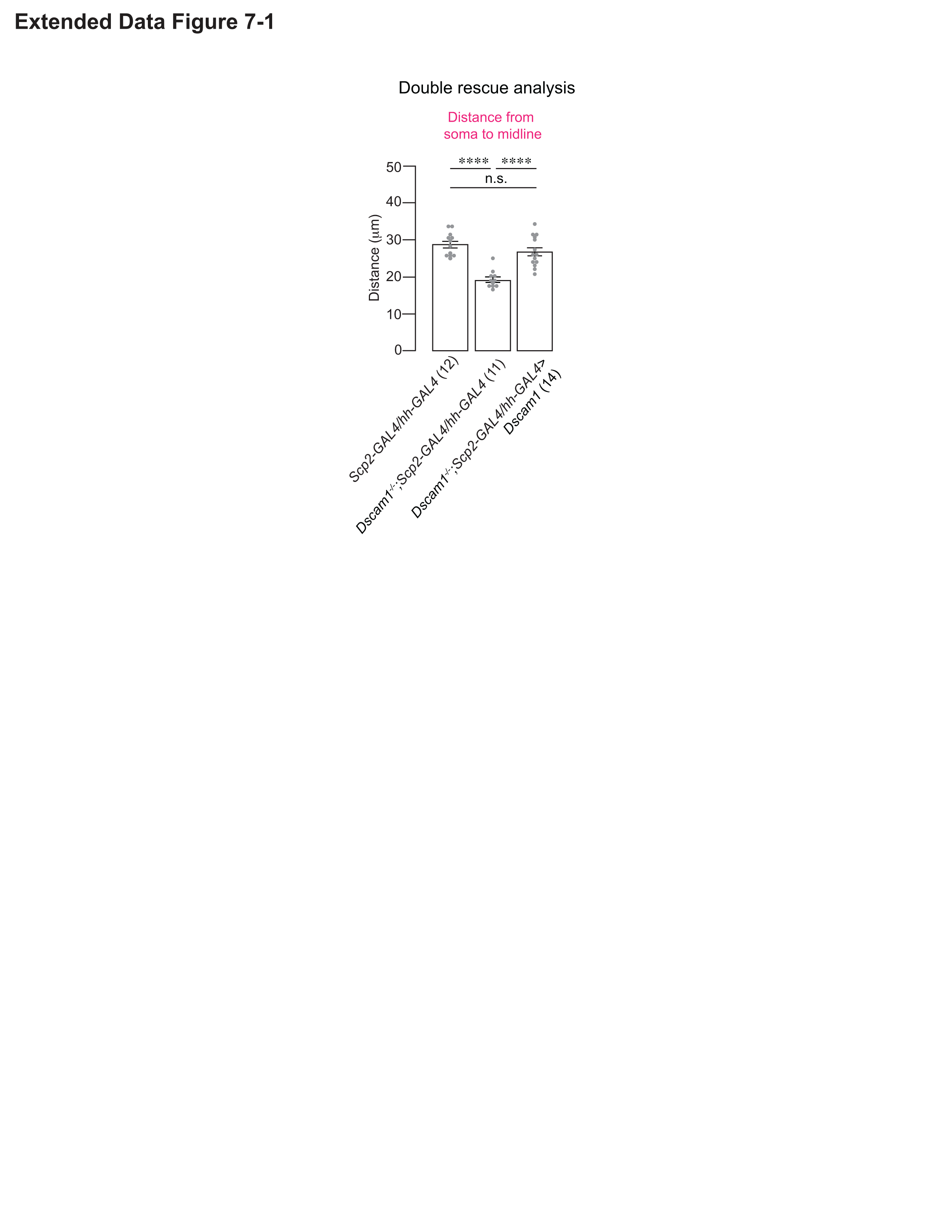

Supplement: Figure 7-1 — Resupplying Dscam1 in Scp2-Positive Lateral Fascicle and MN24 Restores Mutant MN24 Soma Position Quantification of MN24 soma positions in wild-type background with Scp2- and hh-specific expression of membrane-bound GFP, Dscam1-/- mutant background with Scp2- and hh-specific expression of membrane-bound GFP, and Dscam1-/- mutant background with combined Scp2- and hh-specific resupply of Dscam1; using Kruskal–Wallis test followed by Dunn’s multiple comparisons test. Download Figure 7-1, TIF file. [file eneuro-11-ENEURO.0130-24.2024-s005.tif]
